# Sustainable trans-scale fibrous membranes for stable ultra-protective air filtration

**DOI:** 10.1038/s41467-026-74514-x

**Published:** 2026-06-23

**Authors:** Yuchen Yang, Xiangshun Li, Yukui Gou, Yunna Hao, Tianxue Zhu, Yuekun Lai, Jianying Huang

**Affiliations:** 1 Fujian Key Laboratory of Functional Textile Fibers and Products, Minjiang University, Fuzhou, PR China; 2 College of Chemical Engineering, Fuzhou University, Fuzhou, PR China; 3 Qingyuan Innovation Laboratory, Quanzhou, PR China; 4 College of Materials and Chemistry, Anhui Agricultural University, Hefei, PR China; 5 College of Materials Science and Engineering, Fuzhou University, Fuzhou, PR China

**Keywords:** Chemical engineering, Structural properties, Mechanical properties, Pollution remediation

## Abstract

Disposable fibrous air filtration media represent one of the most convenient and effective approaches for personal health protection. Nevertheless, restricted by the technical bottlenecks of spinning technologies in fabricating ultrafine nanofibers and designing topological filtration networks, current filters cannot realize reliable ultra-protection against ultrafine particulate matter (PM). Moreover, non-degradable waste from excessive filter use imposes severe environmental burdens. Herein, we report a universal multi-level splitting electrospinning strategy for fabricating a variety of biodegradable trans-scale fiber membranes (TSFM). The hierarchical interweaving and stacking of ultrafine nanofibers, nanofibers, and submicron fibers endow the lightweight TSFM with tortuous interception networks and abundant mass and energy transport channels. Consequently, they can achieve a stable ultra-efficient PM_0.3_ removal, low air resistance, great breathability, and reduce polymer consumption by 99% relative to commercial N95 respirators. This work may boost the iterative upgrading of advanced spinning technologies and the development of high-performance, sustainable filtration and separation materials.

## Abstract (web summary)

Fibrous air filtration media are promising for personal protection, though fabricating ultrafine nanofibers and designing topological filtration networks are challenging. Here the authors report an electrospinning strategy to design a particulate matter filtration membrane.

## Introduction

According to recent statistics from the World Health Organization (WHO), air pollution currently disturbs billions of the world’s population and annually causes millions of premature deaths^1^. Among various airborne pollutants, the inhalable ultrafine aerosols (PM_0.3_, PM_0.5_, PM_1.0_) can not only penetrate and attack human organs, but also carry pathogenic germs and viruses for pandemic transmission^2^. As a result, they have repeatedly caused severe respiratory occupational diseases (e.g., asthma, pneumoconiosis, tuberculosis), infectious diseases (e.g., influenza), and even global pandemics (e.g., COVID-19), thus emerging as one of the gravest environmental threats to human health and public safety^3,4^. Among various air filtration systems, fibrous filters are the most effective, mature, and practical personal protective pathways due to their distinctive structural characteristics^4–7^. They are stacked by the multilayer melt-blown or glass ultrafine fibers (over 1 μm) and thus possess the characteristics of micro-scale pores, high areal density (>50 g/m^−2^) and considerable thickness (>100 μm)^8–10^. Nevertheless, they suffer from insufficient protective capacity below 99% to the most permeable PM_0.3_^8,9^. The bulky filter structure severely impedes the transfer of airflow, moisture, and heat, which significantly compromises wearing comfort^11^. Besides, disposable air filters with high base weight consume large quantities of raw materials, and they even led to acute supply shortages during the epidemic period^12,13^. Meanwhile, the massive discard of non-degradable filters in that period also generated over 8 million tons of persistent plastic waste, which in turn endangers terrestrial ecosystems, marine environments, and living organisms^12–14^. Consequently, the development of advanced air filters that can simultaneously improve protective performance, wearing comfort, and sustainability is urgently required.

Air filters made of finer fibers typically exhibit better properties, including a higher specific surface area, greater porosity, smaller pore size, and so on^15–17^. These characteristics not only enhance the filtration efficiency against PM_0.3_ but also enable the reduction of filter thickness and areal density^17^. The resultant lightweight and thin features are also conducive to the sustainability and wearing comfort of such air filters^11,17^. Accordingly, extensive research efforts have been devoted to refining fiber diameters down to the submicron scale via various innovative techniques, such as laser-assisted melt-blown spinning^17^, solution blow spinning^18^, centrifugal spinning^19^, sea-island spinning^20^, electrospinning^21–23^, and so forth^24,25^. Notably, nano-scale fibers (<100 nm) can be even prepared by facilitating jet stretching through designing the electrospun precursor solutions or spinnerets^26,27^. The prepared nanofiber filters achieve efficient PM_0.3_ removal (>99%), coupled with low thickness and base weight. But the airflow drag effect induced by nanofibers and their densely packed structure also leads to unsatisfactory filtration resistance^26–28^. Fortunately, the assembly of multi-scale fibers can not only retain the high-efficiency PM_0.3_ capture capability of fine nanofibers, but also optimize airflow channels via the incorporation of coarser fibers, thereby reducing filtration resistance^29–31^. To resolve the trade-off between filtration efficiency and resistance, a variety of thin, lightweight multi-scale fiber filters have been developed, fabricated via self-assembly^32^, electro-netting^33–35^, chemical vapor deposition^36,37^, small molecule mutual support^38^, and phase separation strategies^39^. In our previous work, the designed dual-scale fiber mats, composed of ultrafine nanofibers (<50 nm) and submicron fibers, can intercept over 99.9% PM_0.3_ and are comfortable^40^. Beyond their filtration performance, these efficient air filters can reduce the consumption of petroleum-derived polymers by over 80%. However, they are non-biodegradable and can still generate waste pollution^41^. To further enhance the sustainability and comprehensive filtration performance of air filters, most recently, some bio-based polymers, such as polylactic acid (PLA) and cellulose, were utilized to fabricate biodegradable ultrafine nanofiber-based membranes for high-performance air filtration^42,43^. Despite these progress, nevertheless, a universal preparation strategy of producing uniform ultrafine nanofibers and constructing controllable trans-scale fiber interception networks is still blank. Therefore, the development of the biodegradable, ultra-lightweight (≤0.5 g/m^2^), stable ultra-protective (≥99.999% PM_0.3_ removal), and super-comfortable PM_0.3_ air filters remains a bottleneck.

Herein, we report a general multi-level jet-splitting electrospinning strategy for the scalable fabrication of trans-scale fibrous membranes (TSFM) for the construction of the ultra-protective, super-comfortable, and sustainable air filters (Supplementary Fig. S1). Through polyelectrolyte-mediated regulation of precursor solutions, the conventional single jet is converted into multi-level splitting jets, including the main jet, coarse splitting jet, and fine splitting jet via a one-step electrospinning process. Under the effect of a high-voltage electrostatic field, these jets further evolve into submicron fibers (>100 nm), nanofibers (<100 nm), and ultrafine nanofibers (<50 nm), respectively. Furthermore, this strategy is compatible with a diverse range of bio-based polymers, namely (polybutylene succinate, PBS; PLA; Polyamide 56, PA56), enabling the preparation of biodegradable TSFM. The hierarchical stacking architecture of trans-scale fibers endows the filtration media with an ultralow pore diameter, a high specific surface area, curved interception networks, and abundant interconnected transfer channels. Consequently, the three types of TSFM prepared via this approach exhibit ultra-protective performance, achieving over 99.999% removal efficiency for ultrafine PM along with excellent stability throughout multiple filtration cycles or at different airflow rates. These air filters also deliver great wearing comfort, characterized by low airflow resistance, high breathability, favorable vapor permeability, and efficient heat dissipation. Moreover, the ultra-lightweight and biodegradable TSFM can reduce polymer consumption by more than 99% while retaining their ultra-protective capacity and comfort level.

## Results

### The design of TSFM filters

**Fig. 1:**
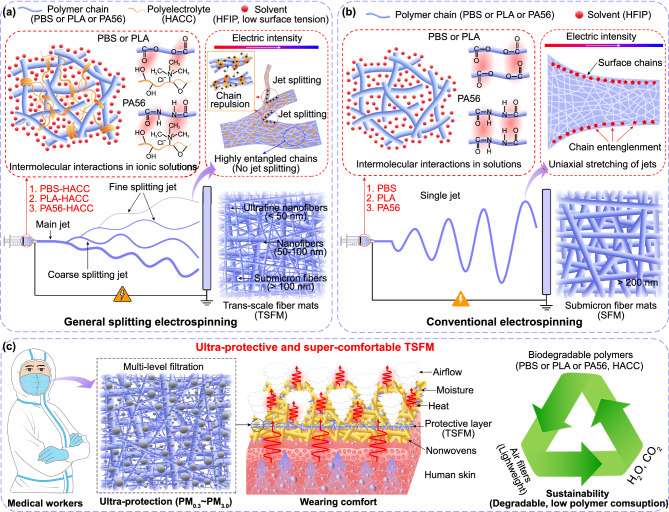
Formation principle, features, and applications of TSFM and SFM. **a** Schematic diagram of TSFM prepared by the general multi-level splitting electrospinning strategy. **b** Schematic illustration of SFM prepared by the conventional electrospinning strategy. **c** Schematic demonstration of the ultraprotective, supercomfortable, and sustainable characteristics of TSFM.

To ensure the environmental friendliness of the filtration media, three types of bio-based polymers (Polybutylene succinate, PBS; Polylactic acid, PLA; Polyamide 56, PA56) were selected as the raw materials for preparing disposable face masks (Fig. 1a). These polymers contain the functional group of −COO− (e.g., PBS, PLA) or −CO−NH− (e.g., PA56), and can also dissolve in HFIP to form a homogeneous solution. Due to the strong hydrogen bonding interactions and entanglement between macromolecular chains, the macromolecular chains on the surface of the jet are constrained by the molecular chains inside the jet during the conventional electrospinning process (Fig. 1a). As a result, a single jet is formed, which ultimately evolves into submicron fibers with diameters exceeding 200 nm owing to the limited uniaxial stretching effect (Fig. 1b).

Therefore, we proposed a general one-step multi-level splitting electrospinning technique to simultaneously refine fiber diameter and construct TSFM (Fig. 1a, Supplementary Fig. S1). A precursor solution system, composed of bio-based polymers, charged cationic polyelectrolytes, and low surface tension solvents, was designed to lay the foundation for the multi-level jet splitting. Hydroxypropyl trimethylammonium chloride chitosan (HACC) possesses multiple functional groups, including −OH, −C−O−C−, and [N(CH_3_)_3_]^+^. It can interact with bio-based polymers through hydrogen bonding and electrostatic forces between their respective functional groups, and uniformly segment the entangled macromolecular chain networks. Meanwhile, it can also dissociate into charged cations in solvents, which induces strong electrostatic repulsion between polymer chains. As is widely recognized, the formation of electrospun jets arises from the electrostatic field force overcoming the surface tension of the solution. The two bulky trifluoromethyl groups (-CF_3_) in HFIP possess low polarizability and strong steric hindrance, which drastically weaken intermolecular forces and cohesive forces between liquid molecules^44^. The introduction of hexafluoroisopropanol (HFIP) can effectively decrease the surface tension of precursor solutions and thus facilitate the multi-level jet splitting. Finally, the main jet, secondary coarse splitting jet, and fine splitting jet evolve into submicron fibers (>100 nm), nanofibers (50–100 nm), and ultrafine nanofibers (<50 nm), respectively.

The ultrafine fiber diameter and hierarchical membrane architecture endow the TSFM with multi-level filtration characteristics and ultra-protective capacity against PM_3.0_ and even ultrafine PM_0.3_ (Fig. 1c). Moreover, the ultrathin membrane structure also decreases the transport resistance and thus promotes the rapid transfer of both airflow, heat, and moisture. Therefore, the distinctive membrane design effectively balances the personal protection and wearing comfort of the filter media. The ultra-protective and super-comfortable TSFM filters are anticipated to be widely utilized as face masks by the public for daily protection (Fig. 1c). Meanwhile, the lightweight feature of the filtration media can not only significantly reduce the polymer consumption for the preparation of protective filters, but also strongly alleviate the supply shortage of such filters during outbreaks of respiratory infectious diseases. In addition, the use of bio-based raw materials enables the natural degradation of disposable face masks, making the product environmentally sustainable (Fig. 1c).

### Formation mechanism of trans-scale fibers

**Fig. 2:**
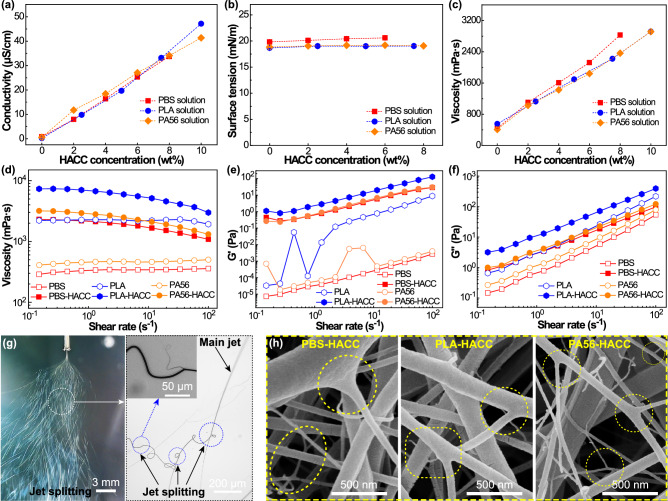
Properties of precursor solutions and evidence of jet splitting. **a** Conductivity, **b** surface tension, and **c** viscosity of precursor solutions with varying HACC concentrations. **d** Viscosity as a function of shear rate for PBS, PLA, and PA56 solutions with HACC and without HACC. **e** Storage moduli (G^’^) of PBS, PLA, and PA56 solutions with HACC and without HACC. **f** Loss moduli (G^”^) of PBS, PLA, and PA56 solutions with HACC and without HACC. **g** Optical images of jet splitting during the electrospinning process. **h** SEM images of different splitting fibers. Error bars represent the standard deviation of the measured properties and the samples are at least three. Source data are provided as a Source Data file.

Solution properties, such as the conductivity, surface tension, and rheological characteristics, determine the jet formation and fiber morphology. As shown in Fig. 2a and Supplementary Fig. S2a, the incorporation of HACC effectively improved the conductivity of three kinds of polymer solutions. With increasing HACC concentration, the solution conductivity increased linearly, which can be attributed to the free ions generated by HACC, such as [N(CH_3_)_3_]^+^ and Cl^-^. As shown in Supplementary Fig. S2, the conductivity of PBS/HFIP, PLA/HFIP, and PA56/HFIP solutions was nearly zero, whereas HACC/HFIP solutions with different ratios exhibited the highest conductivity. Notably, the conductivity of ternary solutions containing polymer, HACC, and HFIP was significantly lower than that of binary HACC-HFIP solutions (Supplementary Fig. S2a). It demonstrated that the introduction of polymers such as PBS, PLA, and PA56 significantly decreased the solution conductivity. This result indicates that polymer-HACC interactions hinder the migration of charged ions.

Owing to the use of HFIP as a solvent, all polymer solutions with varying HACC concentrations exhibited low surface tension (Fig. 2b and Supplementary Fig. S2b). This feature effectively lowers the jet formation threshold, thereby creating favorable conditions for promoting jet splitting. The viscosity of the solutions was positively correlated with HACC concentration (Fig. 2c and Supplementary Fig. S2c), which is indicative of intermolecular interactions and chain entanglement between HACC and the polymers^45^. As illustrated in Fig. 2d, pure polymer solutions exhibited stable entangled chain networks. In contrast, HACC-doped polymer solutions displayed shear-thinning behavior over a shear rate range of 10^−1^ to 10^2^ s^−1^ (Fig. 2d). Notably, the viscosity of HACC-doped solutions was higher than that of pure polymer solutions. These results suggest that the shorter HACC chains have undergone orientation under shear stress, while also verifying the strong interactions between HACC and different polymers. Moreover, in comparison to pristine solutions, both storage moduli (G^’^) and loss moduli (G^”^) of solutions doped with HACC were elevated (Fig. 2e, f), manifesting the formation of stable HACC-polymer chain aggregates^46^.

The designed supramolecular assembly, consisting of polymer, HACC, and HFIP, possesses a high density of charged ions and exhibits low surface tension. In addition, HACC acts as a molecular spacer to separate polymer chains, thereby facilitating the formation of charged aggregates that exert mutual repulsion under a high-voltage electrostatic field. During the electrospinning process, multiple secondary jets and fine jets are generated from the main jet and secondary jets, respectively (Fig. 2g). A trans-scale fibrous structure was observed across all three distinct polymer solution systems, in which coarse fibers branch into hierarchical fine fibers (Fig. 2h). Therefore, the tailored supramolecular assembly induces the asymmetric multi-level jet splitting that a primary initial jet transforms into numerous secondary and fine jets. These branched jets exhibit a diameter gradient, enabling the fabrication of trans-scale fibers with a diameter gradient. Spinning solutions with high conductivity, low surface tension, and shear-thinning behaviors contribute to the jet splitting^47^.

### Structural and mechanical properties of TSFM filters

**Fig. 3:**
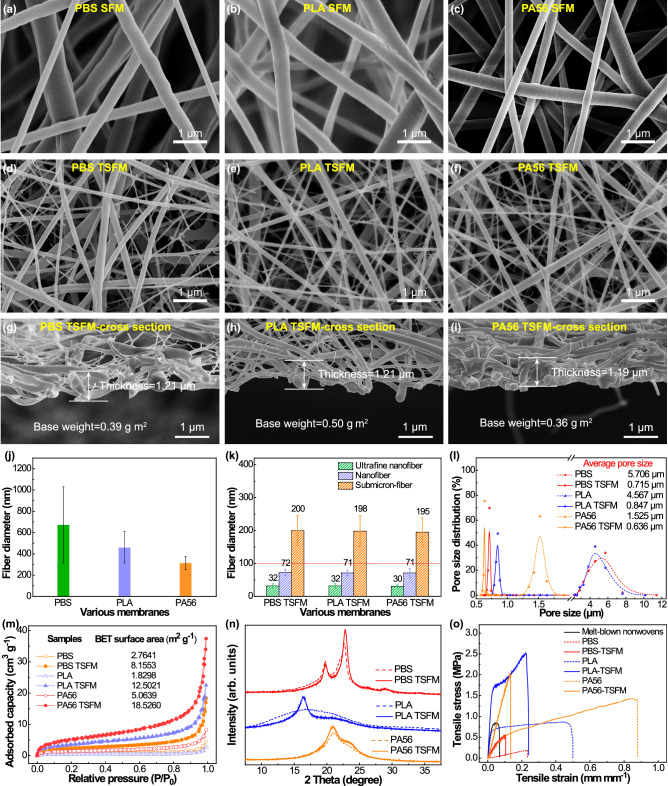
Morphologies, structures, and mechanical properties of various fibrous membranes. SEM images of **a** PBS SFM, **b** PLA SFM, **c** PA56 SFM, **d** PBS TSFM, **e** PLA TSFM, and **f** PA56 TSFM. Cross-sectional SEM images of **g** PBS TSFM, **h** PLA TSFM, and **i** PA56 TSFM. **j** Fiber diameter of PBS SFM, PLA SFM, and PA56 SFM. **k** Diameters of ultrafine nanofibers, nanofibers, and submicron fibers in PBS TSFM, PLA TSFM, and PA56 TSFM. **l** Pore size distributions of various SFM and TSFM. **m** BET isotherms of various SFM and TSFM. **n** XRD patterns of various SFM and TSFM. **o** Tensile stress-strain curves of the various fibrous membranes. Error bars represent the standard deviation of the measured properties and the samples are at least thirty. Source data are provided as a Source Data file.

As shown in Fig. 3a-c, the diameters of electrospun PBS, PLA, and PA56 fibers were at the submicron scale (>200 nm). In contrast, the incorporation of HACC into the precursor solutions induced jet splitting during electrospinning, yielding ultrafine fibers with gradient diameter distributions (Supplementary Figs. S3–5). Furthermore, increasing the HACC content facilitated jet splitting and thus promoted fiber diameter refinement. However, excessive HACC addition compromised the spinnability of the solutions owing to the resultant high viscosity. As depicted in Fig. 3d–f, three types of uniform TSFM were fabricated from 10 wt%PBS/6 wt%HACC, 8 wt%PLA/7.5 wt%HACC, and 6 wt%PA56/8 wt%HACC solutions, respectively. As illustrated in Fig. 3g–i, the as-prepared TSFM featured an ultrathin, porous membrane architecture with curved, highly interconnected pore channels. For comparison, the diameters of commercial melt-blown fibers exceeded 1 μm, whereas those of electrospun bio-based fibers ranged from 200 nm to 1 μm (Fig. 3j and Supplementary Fig. S6). By contrast, TSFM were composed of a hierarchical fiber network, including submicron fibers (100–300 nm), nanofibers (50–100 nm), and ultrafine nanofibers (0–50 nm), exhibiting a substantially finer fiber diameter profile (Fig. 3k).

The structural characteristics of TSFM were further investigated. The pore size of electrospun fiber mats exhibited a positive correlation with HACC concentration (Supplementary Fig. S7). The pore size and specific surface area of the membrane are positively and negatively correlated with the fiber diameter, respectively^48–50^. As presented in Fig. 3l, PBS, PLA, and PA56 SFM comprising 200-1000 nm submicron fibers possessed pore sizes larger than 1 μm. In contrast, PBS, PLA, and PA56 TSFM were composed of ultrafine nanofibers, nanofibers, and submicron fibers with considerably finer diameters, exhibiting remarkably smaller pore sizes of 0.715, 0.847, and 0.636 μm, respectively. Furthermore, owing to the pronounced reduction in fiber diameter, the BET specific surface area of TSFM was more than threefold higher than that of electrospun PBS, PLA, and PA56 SFM (Fig. 3m). In comparison to electrospun biso-based fibers (PBS, PLA, PA56), TSFM displayed enhanced crystallinity (Fig. 3n). This phenomenon is primarily attributed to HACC-induced jet stretching and the dense packing of aligned polymer chains under the nanoconfinement effect^51^. Consequently, TSFM also exhibited considerably higher tensile strength alongside reduced elongation at break compared with the pristine electrospun fibers (Fig. 3o). Notably, the overall tensile performance of PLA and PA56 TSFM surpassed that of commercial melt-blown nonwovens, while PBS TSFM demonstrated better elongation at break with marginally lower tensile strength. The robust tensile properties of TSFM provide strong support for their practical application in personal protective filtration materials.

### Filtration performance of TSFM filters

**Fig. 4:**
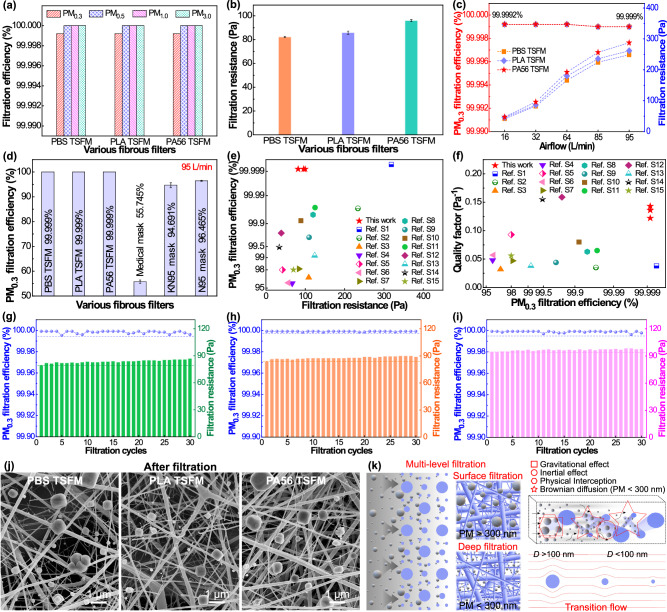
Filtration performance and mechanism of TSFM. **a** Filtration efficiency of various TSFM to PM_0.3_, PM_0.5_, PM_1.0_, and PM_3.0_. **b** Filtration resistance of various TSFM at an airflow rate of 32 L/min. **c** Effect of airflow rate on the filtration efficiency and resistance of TSFM. **d** PM_0.3_ removal efficiency of various air filters at an airflow rate of 95 L/min. **e** Comparison of PM_0.3_ filtration efficiency and resistance between this work and recent reports. **f** Comparison of PM_0.3_ filtration efficiency and quality factor between this work and recent reports. PM_0.3_ filtration efficiency and resistance of **g** PBS TSFM, **h** PLA TSFM, **i** PA56 TSFM during filtration cycling. **j** SEM images of TSFM filters with PM capture. **k** Schematic diagram of the PM filtration mechanism of TSFM. Error bars represent the standard deviation of the measured properties and the samples are at least three. Source data are provided as a Source Data file.

To verify the personal protective performance of the TSFM filters, the PM filtration properties of various air filters were studied (Fig. 4 and Supplementary Figs. S8–12). With increasing HACC concentration in the precursor solutions, the prepared fiber mats exhibited a synchronous increase of PM_0.3_ filtration efficiency and airflow resistance (Supplementary Figs. S8–10). This trend is attributed to the reduced fiber diameter, increased membrane pore size, and elevated specific surface area of the mats. Notably, the substantial enhancement of the quality factor (QF) of the three kinds of bio-based fiber membranes underscores the pivotal role of HACC in optimizing the overall filtration performance of air filters. The PBS, PLA, and PA56 TSFM achieved the maximum QF value of 0.143, 0.136, and 0.122 when HACC concentration was 6 wt%, 7.5 wt%, and 8 wt%, respectively. Meanwhile, all three types of TSFM demonstrated exceptional protective capacity that their PM_0.3_ removal efficiency exceeded 99.999%, and their PM_0.5_, PM_1.0_, and PM_3.0_ capture efficiency reached 100% (Fig. 4a). Nevertheless, their filtration resistance remained below 100 Pa (Fig. 4b). The filtration properties of TSFM filters could also be modulated by tailoring their base weight (Supplementary Figs. S8–10). Specifically, increasing filter base weight boosted the PM_0.3_ capture efficiency from 85% to 99.9992%, thereby enabling customizable performance for diverse air filtration application scenarios.

The filtration stability and durability of air filters were also evaluated. As the airflow rate increased from 16 to 95 L/min, all three kinds of commercial face masks (medical mask, KN95 mask, and N95 mask) exhibited a significant decline in both PM_0.3_ removal efficiency and quality factor (QF), accompanied by a dramatic increase in filtration resistance (Supplementary Fig. S11-12). By contrast, the TSFM maintained efficient and stable PM_0.3_ capture (≥99.999%) and ultra-protective capacity (Fig. 4c). Although the filtration resistance of TSFM filters also sharply increased with rising airflow rate, their QF remained substantially higher than that of commercial air filters (Fig. 4c and Supplementary Fig. S12). As presented in Fig. 4d, medical masks, KN95 masks, and N95 face masks demonstrated poor protective performance against the most permeable particle size (MPPS) of PM_0.3_, with removal efficiencies of only 55.745%, 94.691%, and 96.465%, respectively. In comparison to the latest literature reports, the prepared TSFM filters achieved great overall filtration performance, including over 99.999% PM_0.3_ removal, low air resistance of less than 100 Pa, and high QF of over 0.12 (Fig. 4e, f and Supplementary Table S1). To verify the long-term filtration stability of TSFM, cyclic filtration tests were conducted as illustrated in Fig. 4g–i. After 30 consecutive cycles of filtration evaluation, all TSFM filters retained the ability to intercept over 99.996% of PM_0.3_ while maintaining low filtration resistance (<100 Pa), demonstrating exceptional personal protective performance. During cyclic filtration, the gradual deposition of particulate matter within the filter media partly blocked its pores and narrowed the airflow channels^11,40^. This results in a slight increase in filtration resistance of three TSFM filters.

The filtration mechanism of TSFM filters was further analyzed. In contrast to the surface potential of commercial electret melt-blown fibers (exceeding −1 kV), that of TSFM was close to 0 V, which is attributed to HACC-induced rapid potential decay (Supplementary Fig. S13). This result strongly indicates that commercial filters primarily capture aerosols via electrostatic attraction, whereas TSFM relies on a non-electrostatic filtration mechanism. Moreover, high airflow rates shorten the residence time of particulate matter within air filters, thereby impairing both physical sieving and electrostatic interception of commercial filters. This fundamental difference accounts for the unstable PM capture efficiency of commercial filters and the sustained filtration performance of TSFM filters^37–40^. As depicted in Fig. 4j, fine particles (PM_1.0_, PM_3.0_) were trapped by the submicron pores of TSFM, while the ultrafine particles (PM_0.3_, PM_0.5_) were preferentially captured by nanofibers and ultrafine nanofibers. Therefore, TSFM integrate two synergistic filtration modes of surface filtration and deep filtration, thereby enabling multi-level aerosol interception and ultrahigh protective performance (Fig. 4k). For surface filtration of fine particles, gravitational effect, inertial effect, and physical interception were the dominant avenues. In contrast, ultrafine particles exhibit pronounced Brownian diffusion behavior, with diffusion intensity increasing as particle size decreases^40^. As a consequence, the ultrafine particles with negligible weight and tiny volume were easily intercepted by hierarchical and porous fiber networks through the deep filtration during their diffusion process. Notably, the hierarchical stacking structure and submicron pores of TSFM, consisting of submicron fibers, nanofibers, and ultrafine nanofibers, endow the air filter with a multi-level interception network and thus ultra-protective performance of over 99.999% PM_0.3_ removal. Meantime, trans-scale ultrafine fibers are at the transition flow, and their hierarchical stacking structure also effectively minimizes the drag resistance of airflow^33–35^. Therefore, the trade-off between pressure drop and filtration efficiency was realized by the ultrafine nanofiber preparation and hierarchical structure design of the air filter^33–35^.

### Wearing comfort of the TSFM filter

**Fig. 5:**
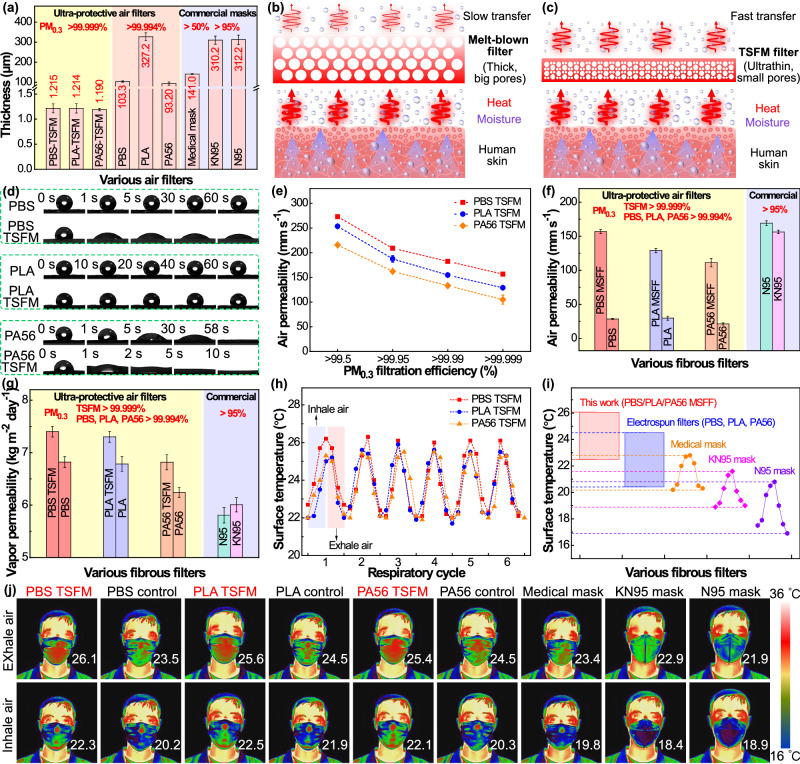
Wearing comfort of TSFM filters. **a** Thickness comparison of various air filters. **b** Schematic diagram of rapid heat and moisture transfer from human skin through TSFM filters. **c** Schematic diagram of slow heat and moisture transfer from human skin through melt-blown fiber filters. **d** Dynamic water contact angle of various fiber membranes. **e** Relationship between air permeability and PM_0.3_ filtration efficiency of various TSFM. **f** Air permeability comparison of various air filters. **g** Vapor permeability comparison of various air filters. **h** Surface temperature changes of various TSFM during continuous respiratory cycles. **i** Surface temperature variation ranges of various TSFM filters and SFM filters during continuous respiratory cycles. **j** Infrared thermography images of various air filters. Error bars represent the standard deviation of the measured properties and the samples are at least three. Source data are provided as a Source Data file.

Human respiratory movements generate substantial amounts of heat and water vapor. Wearing a protective mask impedes the transfer of heat and moisture, thereby inducing physiological discomfort in the human body. As shown in Fig. 5a, commercial melt-blown fiber masks exhibited insufficient protective performance against PM_0.3_ (filtration efficiency <99%) and an excessively high thickness (>140 μm), while PBS, PLA, and PA56 SFM possessed ultrahigh filtration efficiency but still featured a relatively large thickness (>90 μm). In contrast, the TSFM achieved both ultra-thinness (approximately 1.2 μm) and ultrahigh protective performance. These distinct structural differences between TSFM and commercial filters exerted a significant influence on their wearing comfort. As depicted in Fig. 5b, despite their micro-pores, the commercial protective masks with an ultra-thick structure exhibit retarded, even inhibited heat dissipation and moisture transmission, leading to the accumulation of heat and moisture and thus poor wearing comfort^11^. In contrast, the hierarchical stacking architecture of trans-scale fibers endows TSFM filters with a submicron pore size, a high specific surface area, and curved interception networks, ensuring excellent personal protective performance^40,48–50^. Meantime, abundant interconnected transfer channels and ultrathin thickness are favorable for heat and moisture transfer and resolve the trade-off between filtration efficiency and wearing comfort^11,40^. The designed ultrathin TSFM filters not only drastically reduce airflow resistance and shorten the diffusion path within the porous membrane but also facilitate the rapid transfer of heat and moisture from the skin surface to the ambient atmosphere (Fig. 5c).

The introduction of HACC enhanced the hydrophilicity of the TSFM (Fig. 5d). To validate the wearing comfort of TSFM filters, the air permeability, vapor permeability, and thermal transport properties of various air filters were systematically characterized. As shown in Fig. 5e, as the PM_0.3_ removal efficiency of TSFM filters increased from over 99.5% to above 99.999%, their air permeability gradually decreased from more than 200 mm s^−1^ to over 100 mm s^−1^. In contrast, conventional electrospun fiber filters (PBS, PLA, PA56) exhibited great low air permeability at comparable filtration levels, while commercial N95 and KN95 masks demonstrated marginally higher air permeability but significantly good protective performance (Fig. 5f). Furthermore, the water permeability of TSFM filters was considerably higher than that of conventional electrospun fiber filters and commercial masks, confirming their excellent moisture transfer capability (Fig. 5g). During the process of human respiration, the surface temperature of the face masks could directly reflect the heat dissipation capability and was monitored (Fig. 5h-j). The surface temperature of all tested air filters rose initially and then declined during a single expiratory-inspiratory cycle. The maximum and minimum surface temperatures of TSFM filters were above 25 °C and 22 °C, respectively, which were significantly higher than those of other electrospun fiber filters and commercial filter masks. These results demonstrate that the ultrathin, porous TSFM filters simultaneously achieve ultrahigh protective performance and great wearing comfort, characterized by low filtration resistance, excellent breathability, and efficient thermal dissipation.

### Sustainability of TSFM filters

**Fig. 6:**
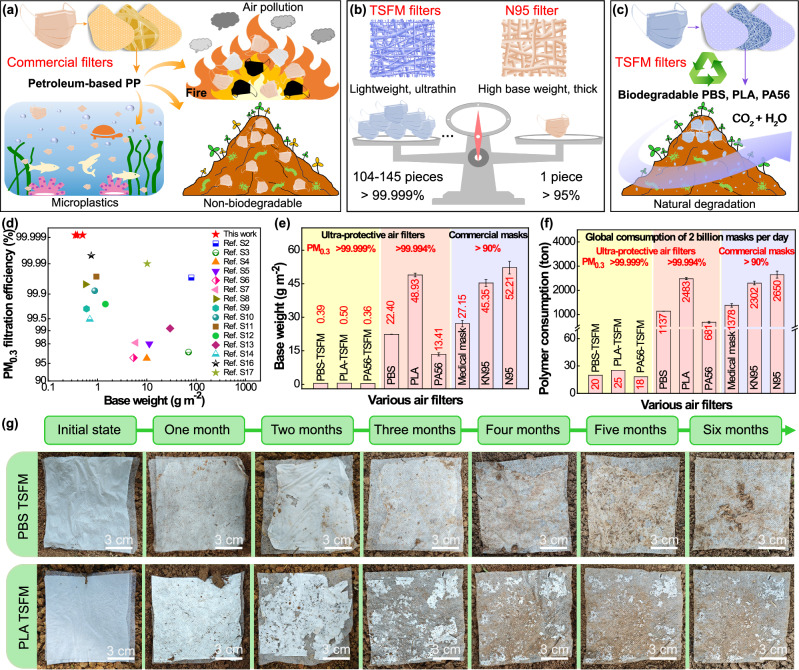
Sustainability evaluation of TSFM filters. **a** Hazard diagram of non-degradable commercial PP filters. **b** Comparison illustration of TSFM filters and N95 filters. **c** Natural degradation diagram of TSFM filters. **d** Comparison of PM_0.3_ filtration efficiency and base weight between our work and recent reports. **e** Comparison of the base weight of various air filters. **f** Polymer consumption for fabricating 2 billion masks using various fibrous membranes. **g** Natural degradation of PBS TSFM and PLA TSFM via soil burial. Error bars represent the standard deviation of the measured properties and the samples are at least three. Source data are provided as a Source Data file.

The sustainable design of air filters encompasses two core aspects: (i) The filters feature an ultralow basis weight, which significantly reduces raw material consumption; (ii) The filters are fabricated from biodegradable polymers, thus exhibiting excellent environmental friendliness (Fig. 6a–c). Commercial air filter products (e.g., N95 masks) are manufactured from non-biodegradable petroleum-based polypropylene (PP) and are discarded in large quantities after single use. Direct incineration of these discarded masks inevitably causes severe air pollution, while landfilling them inflicts lasting damage to the ecological environment. Furthermore, disposable PP masks can degrade into microplastics, which pose a threat to microbial survival and human health through bioaccumulation and food chain transmission. To address this sustainability dilemma of commercial air filters, bio-based polymers (PBS, PLA, PA56) were utilized to construct ultrathin nanofiber networks, which not only substantially enhance PM interception capacity but also minimize the base weight of the biodegradable filters. TSFM filters are designed as disposable biodegradable air filters, and their sustainability is achieved through complete degradation in the natural environment rather than recycling or reuse, which is more in line with the actual application scenarios of disposable air filtration materials. These biodegradable TSFM filters can effectively avoid the post-use recycling and offer a secure, economical end-of-life disposal solution aligned with international sustainable development benchmarks^14,25,43^.

Owing to their ultrafine nano-scale diameters, all three ultra-protective TSFM filters exhibited an ultralow base weight of less than 0.5 g m^−2^, which was about 26-145 times lower than that of SFM filters and commercial filters (Fig. 6d). Compared to state-of-the-art reports, the TSFM filters simultaneously achieved the highest PM_0.3_ removal efficiency and the lowest base weight, thus demonstrating exceptional comprehensive performance (Fig. 6e). Additionally, polymer consumption for manufacturing various air filter products was calculated based on a global daily demand of 2 billion masks^40^ (Fig. 6f). Commercial medical, KN95 and N95 face masks would consume 1378, 2302, and 2650 tons of polymer, respectively. By contrast, PBS, PLA, and PA56 SFM filters required 1137, 2483, and 681 tons of polymer while still achieving PM_0.3_ filtration efficiency exceeding 99.994%. For the fabrication of 2 billion ultra-protective TSFM filters, remarkably, only 20, 25, and 18 tons of PBS, PLA, and PA56 polymers were needed, respectively. This translates to a raw material savings of over 99% for TSFM filters relative to commercial N95 masks. PBS and PLA TSFM filters fully derived from bis-based polymers were chosen for the natural degradation experiment (Fig. 6g). Following six consecutive months of soil burial testing, the majority of the TSFM filter area underwent degradation, whereas the PP fiber substrate remained completely intact. These bio-based filters can undergo enzymatic hydrolysis and microbial degradation, and ultimately decompose into CO_2_ and H_2_O under warm and humid natural environmental conditions.

## Discussion

In summary, we have developed a multi-level jet-splitting electrospinning strategy for fabricating sustainable TSFM for personal protection against air pollution. This facile, scalable and general strategy enables the fabrication of TSFM from various bio-polymers via a straightforward one-step preparation process using conventional multi-needle electrospinning equipment. The as-prepared TSFM are composed of the hierarchically stacking of ultrafine nanofibers (<50 nm), nanofibers (<100 nm), and submicron fibers (>100 nm), and are characterized by a hierarchically stacked architecture, an ultralow pore size, a high specific surface area, curved multi-level interception networks, and abundant interconnected transport channels. Consequently, the TSFM possess multiple advantages: (1) Stable ultra-protective performance against ultrafine aerosols. The TSFM filters exhibit 99.999% PM_0.3_ filtration efficiency and 100% PM_0.5_, PM_1.0_, and PM_3.0_ filtration efficiency after multiple filtration cycles. (2) Great wearing comfort. Despite maintaining an ultra-protective level of 99.999% PM_0.3_ removal, the ultrathin TSFM feature low filtration resistance (<100 Pa), high air permeability (>110 mm s^−1^), and high water vapor transmission rate (>6.8 kg/m^2^/day). (3) Excellent environmental friendliness and sustainability. The TSFM are fabricated from biodegradable polymeric raw materials, and their ultralow areal density (≤0.5 g/m^2^) reduces polymer consumption by up to 99%. This proposed strategy is expected to drive the iterative upgrading of advanced electrospinning technologies, while the as-prepared TSFM may facilitate the sustainable development of next-generation filtration and separation materials and effectively alleviate the white pollution caused by disposable personal protective materials.

## Methods

### Materials

Polybutylene succinate (PBS, Mw: 200,000), polylactic acid (PLA, Mw: 80,000), chitosan quaternary ammonium salt (HACC, degree of substitution: 98%), 1,1,1,3,3,3-Hexafluoro-2-propanol (HFIP, 99.5%) were purchased from Shanghai Macklin Biochemical Technology Co., Ltd. PA56 was acquired from Cathay Biotech Inc. Commercial masks (Medical mask, KN95 mask, N95 mask) were purchased from Cofoe Medical Technology Co., Ltd. The above chemicals were utilized without further purification.

### Preparation of fiber membranes

Firstly, add HACC to HFIP and stir the mixed solutions for 2 h using a magnetic stirrer. Then, various precursor solutions were prepared by dissolving bio-based polymers (PBS or PLA or PA56) in HACC/HFIP solutions after continuous stirring for 12 hours. Afterwards, the commercial multi-needle electrospinning machine (HZ-11, Qingdao Nuokang Environmental Protection Technology Co., Ltd), containing a high-voltage generator, a precise solution supply pump, a rotary drum, and a automatic traverse device, was used to prepare fiber filters. The precise solution supply pump was fixed on the automatic traverse apparatus for uniform deposition of electrospun fibers. The precursor solution was loaded into a syringe with an 18-gauge blunt-tip needle. The details about solution preparation and electrospinning parameters were summarized in Supplementary Table S2. The temperature and relative humidity of the spinning environment were 25 ± 5 °C, 60% ± 5%, respectively.

### Filtration test

According to the Chinese standard (GB13554-2020), the PM filtration efficiency and pressure drop of the air filters were measured by an air filter comprehensive performance tester (LZC-K1, Suzhou Huada Co., Ltd., China). It can produce the charge-neutralized monodisperse solid NaCl aerosols, including PM_0.3_, PM_0.5_, PM_1.0_, and PM_3.0_ from a 2 wt% NaCl aqueous solution. The test area of the air filter was 100 cm^2^, and each sample shall be tested no less than three times. The PM filtration efficiency and resistance of the test air filters were tested under a constant airflow rate of 32 L min^−1^. The quality factor (QF, Pa^−1^) was used to evaluate the comprehensive filtration performances, which can be calculated by the following formula:

1 $${QF}=-{{{\mathrm{ln}}}}(1-\eta ){(\Delta P)}^{-1}$$

where η is the PM filtration efficiency, and ΔP is the filtration resistance. To study the effect of airflow rate on the filtration properties of air filters, the airflow rate was set as 16, 32, 64, 85, and 95 L min^−1^, respectively.

### Characterization

The conductivity, surface tension, and viscosity of precursor solutions were tested by a conductivity meter (FE38, Mettler Toledo Instruments Co., Ltd., Switzerland), a viscometer (LV-SSR, Shanghai Fangrui Instrument Technology Co., Ltd., China), and a surface tension meter (K100, KRÜSS Scientific Instruments Co., Ltd, Germany), respectively. The rheological measurements of the precursor solutions were performed using a rheometer (MCR 302, Anton Paar, Austria) under steady shear and dynamic oscillatory modes. The viscoelastic performances of the precursor solutions were characterized by evaluating the viscosity, storage, and loss moduli as a function of the frequency at 0.1 to 100 rad s^−1^. The optical images of electrospun jets and fiber mats were taken by a mobile phone. A segment of the split jet is intercepted by a rapidly moving glass slide oriented perpendicular to the direction of jet motion, and the morphology of the split jet is observed under an optical microscope. The morphologies of fiber membranes were observed by field emission scanning electron microscopy (S-4800, Hitachi, Japan). The diameter of 50 fibers was tested using the Nano Measurer software. The pore size distribution of fiber mats was studied by a capillary flow porometer (POROLUXTM 100 FM, IB-FT, Germany). The BET surface area of eletrospun fiber mats was determined by an automatic high-performance specific surface area and pore size analyzer (BSD-660-M, Beijing Beishide Instrument Co., Ltd., China). Crystalline structure of electrospun fibers was determined by a powder X-ray diffraction (XRD) diffractometer (Rigaku D/max Ultima IV, Japan). Tensile mechanical properties of fiber membranes were examined by a multi-functional electronic fabric tester (HD026S−100, Nantong Hongda Experimental Instrument Co., Ltd., China) with a clamping distance of 10 cm. The membrane thickness was assessed five times by a digital micrometer thickness gauge. The base weight of the membrane was obtained by quantifying its mass per unit area using an electronic balance (BSA224S, Beijing Sadolis Scientific Instrument Co., Ltd., Germany). An integrated digital goniometer (OCA25, Dataphysics Instruments Co., Ltd., Germany) was employed to reveal the dynamic water contact angle of membranes. A permeability tester (YG461E-III, Wenzhou Fangyuan Instrument Co., Ltd., China) was used to measure the air permeability of air filters. A moisture permeability analyzer (FX3180-CM15, TEXTEST, Switzerland) was utilized to investigate the water vapor transmission rate (WVTR). The surface potential of air filters was assessed by an electrostatic field meter (FMX-003, KREVOR Co., Ltd., China). The thermal images of filters worn by a volunteer (First author, male, 31 years old) were recorded by an infrared camera (HMTPK20-3AQF/W, Hangzhou Hikvision Electronics Co., Ltd., China). The written and informed consent was received from the volunteer. The images (Fig. 1, Fig. 4k, Fig. 5a–c, Fig. 6a–c,) were created by the first author using Paint 3D software and PowerPoint.

### Biodegradability

PBS and PLA are well-recognized biodegradable polymers^49,52^. To evaluate the degradation behavior of TSFM fabricated from these bio-polymers under real natural conditions, the above fibrous samples were randomly buried in soil on the campus of Fuzhou University for natural degradation. PBS and PLA TSFM with an area of 100 cm^2^ and a weight of about 0.05 g were placed on the surface of commercial polypropylene fiber nonwovens. The degradation behavior of the buried composite membranes was monitored periodically, with optical images captured at fixed intervals for characterization.

## Supplementary information

Supplementary Information

Transparent Peer Review file

## Source data

Source Data

## Data Availability

The data that supports the findings of the study are included in the main text and supplementary information files. All data are available from the corresponding author upon request. Source data are provided with this paper.
