# Supplementary material for: Sustainable trans-scale fibrous membranes for stable ultra-protective air filtration
Source: Nat Commun. 2026 Jun 23;17:7876. doi: 10.1038/s41467-026-74514-x (PMC13443542; doi:10.1038/s41467-026-74514-x)
Supplement: Supplementary file 1 [file 41467_2026_74514_MOESM1_ESM.pdf]

Supplementary Information

Sustainable trans-scale fibrous membranes for stable ultra-protective air filtration

Yuchen Yang[1, 2, 3], Xiangshun Li[1], Yukui Gou[2], Yunna Hao[1], Tianxue Zhu[4, *], Yuekun Lai[2, 3, 5*], Jianying Huang[2, *]

[1] Key Laboratory of Novel Functional Textile Fibers and Products, Minjiang University, Fuzhou 350108, P. R. China

[2] College of Chemical Engineering, Fuzhou University, Fuzhou 350108, P. R. China

[3] Qingyuan Innovation Laboratory, Quanzhou 362801, P. R. China

[4] College of Materials and Chemistry, Anhui Agricultural University, Hefei 230036, P. R. China

[5] College of Materials Science and Engineering, Fuzhou University, Fuzhou 350108, P. R. China

[*]Corresponding Author: E-mail: txzhu@ahau.edu.cn; yklai@fzu.edu.cn; jyhuang@fzu.edu.cn

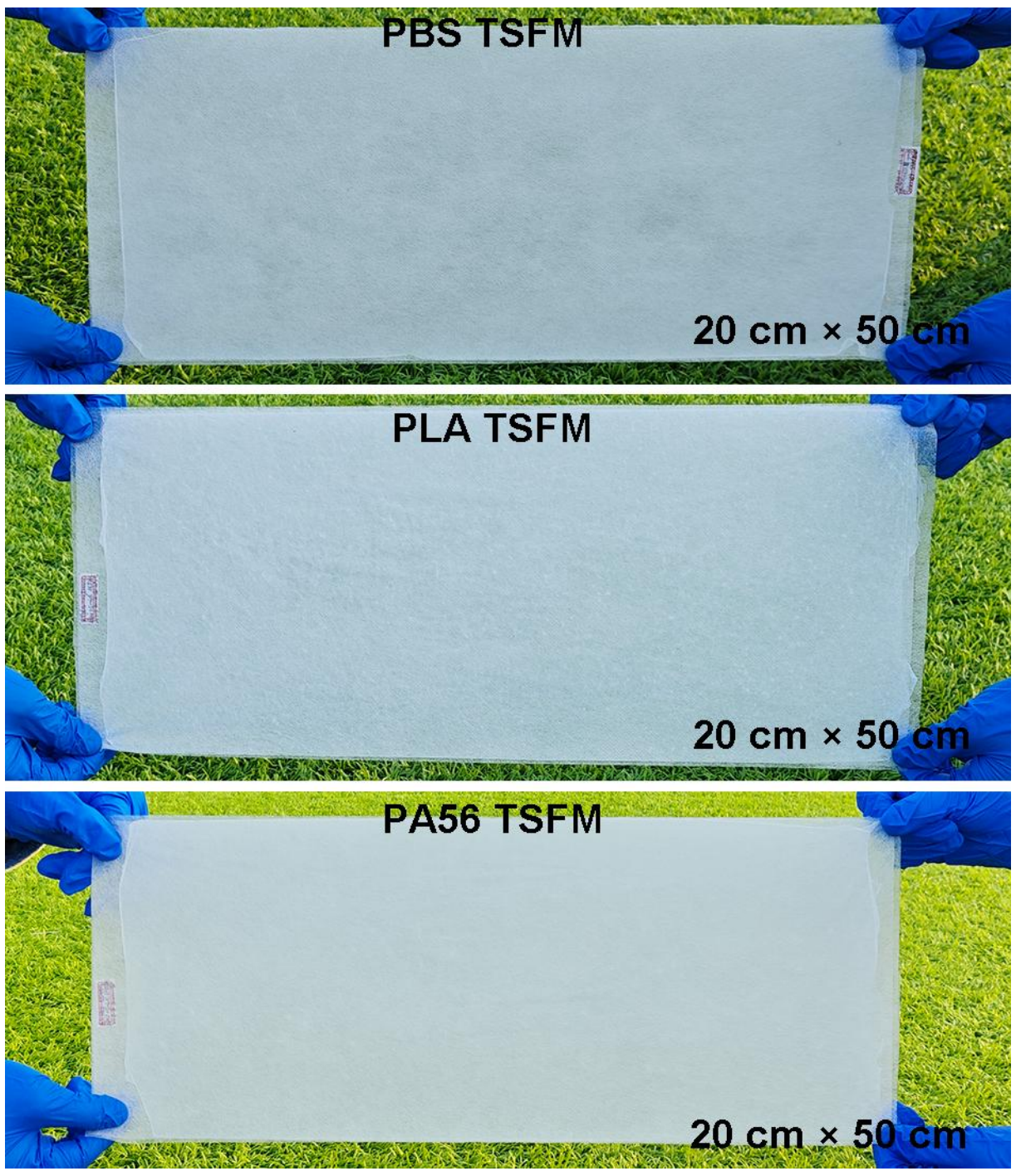

Fig. S1 Optical images of the PBS TSFM, PLA TSFM, and PA56 TSFM.

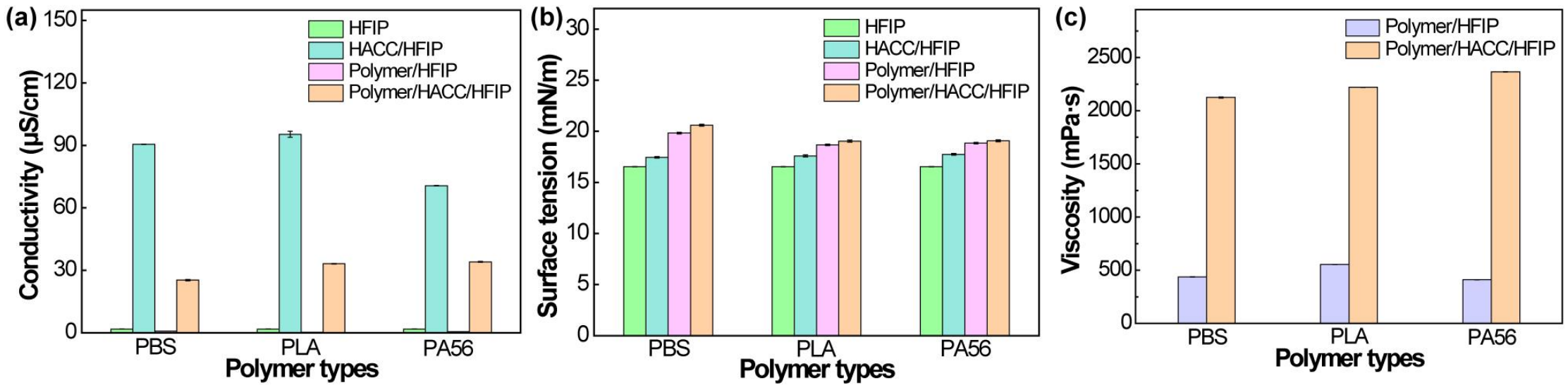

Fig. S2 Properties of different solutions. (a) Conductivity of HFIP, HACC/HFIP, polymer/HFIP, and polymer/HACC/HFIP solutions. (b) Surface tension of HFIP, HACC/HFIP, polymer/HFIP, and polymer/HACC/HFIP solutions. (c) Viscosity of polymer/HFIP and polymer/HACC/HFIP solutions. Error bars represent the standard deviation of the measured properties and the samples are at least three. Source data are provided as a Source Data file.

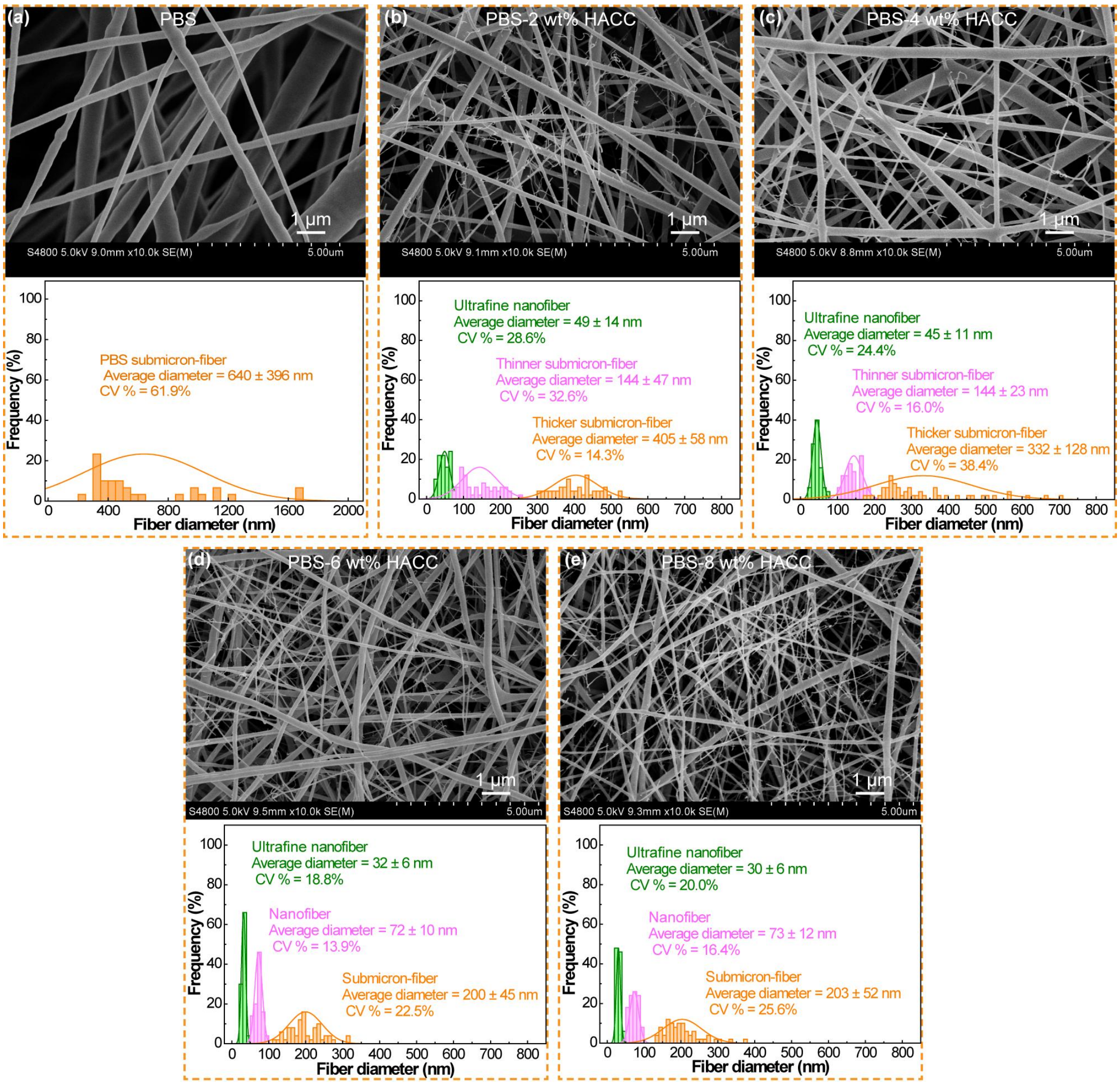

Fig. S3 Morphological characteristics of different PBS fiber mats. (a) SEM images and fiber diameter distributions of mats prepared from 10 wt% PBS solution. (b) SEM images and fiber diameter distributions of mats prepared from 10 wt% PBS/2 wt% HACC solution. (c) SEM images and fiber diameter distributions of mats prepared from 10 wt% PBS/4 wt% HACC solution. (d) SEM images and fiber diameter distributions of mats prepared from 10 wt% PBS/6 wt% HACC solution, (e) SEM images and fiber diameter distributions of mats prepared from 10 wt% PBS/8 wt% HACC solution. Error bars represent the standard deviation of the measured properties and the samples are at least thirty. Source data are provided as a Source Data file.

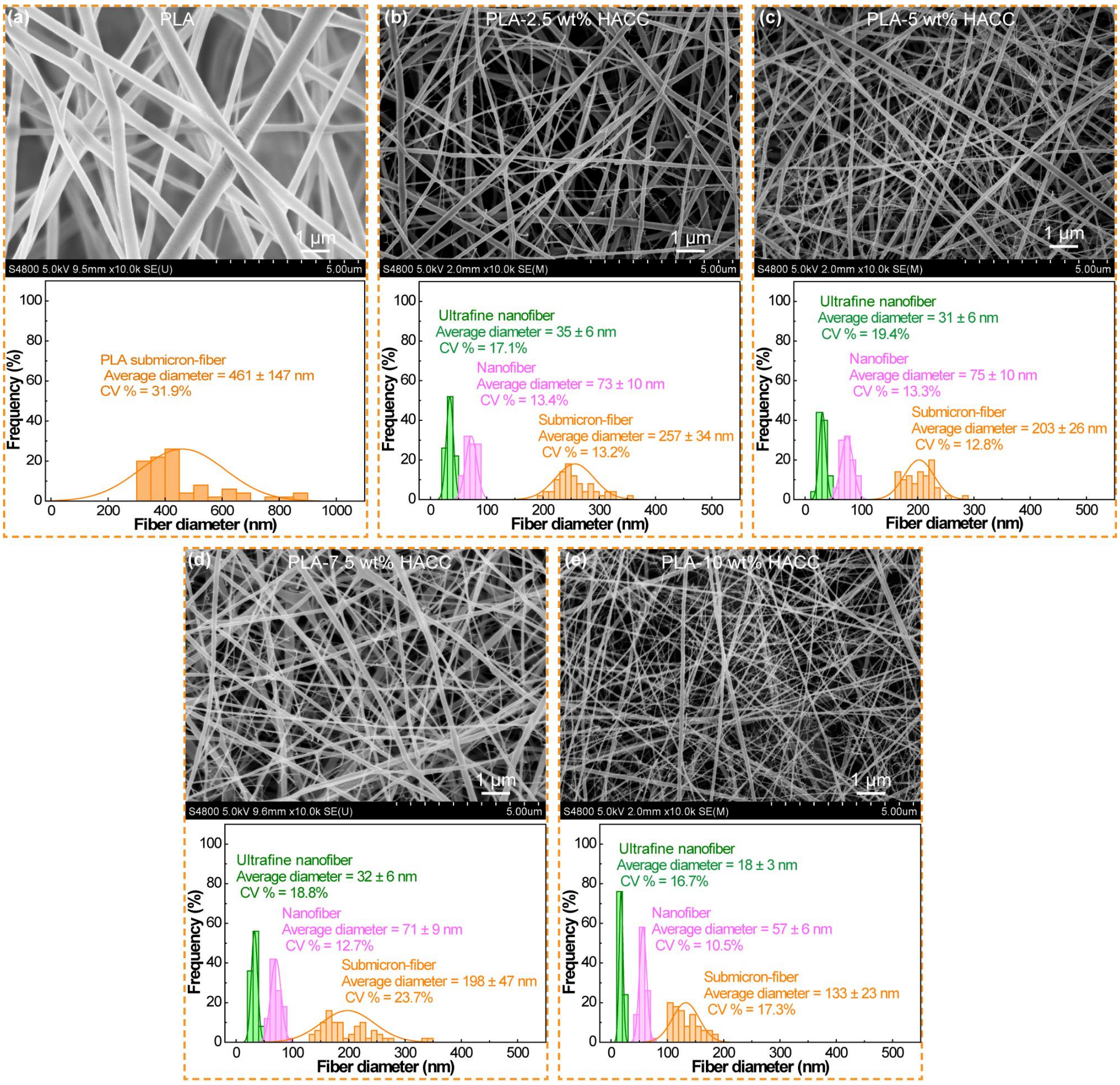

Fig. S4 Morphological characteristics of different PLA fiber mats. (a) SEM images and fiber diameter distributions of mats prepared from 8 wt% PLA solution. (b) SEM images and fiber diameter distributions of mats prepared from 8 wt% PLA/2.5 wt% HACC solution. (c) SEM images and fiber diameter distributions of mats prepared from 8 wt% PLA/5 wt% HACC solution. (d) SEM images and fiber diameter distributions of mats prepared from 8 wt% PLA/7.5 wt% HACC solution. (e) SEM images and fiber diameter distributions of mats prepared from 8 wt% PLA/10 wt% HACC solution. Error bars represent the standard deviation of the measured properties and the samples are at least thirty. Source data are provided as a Source Data file.

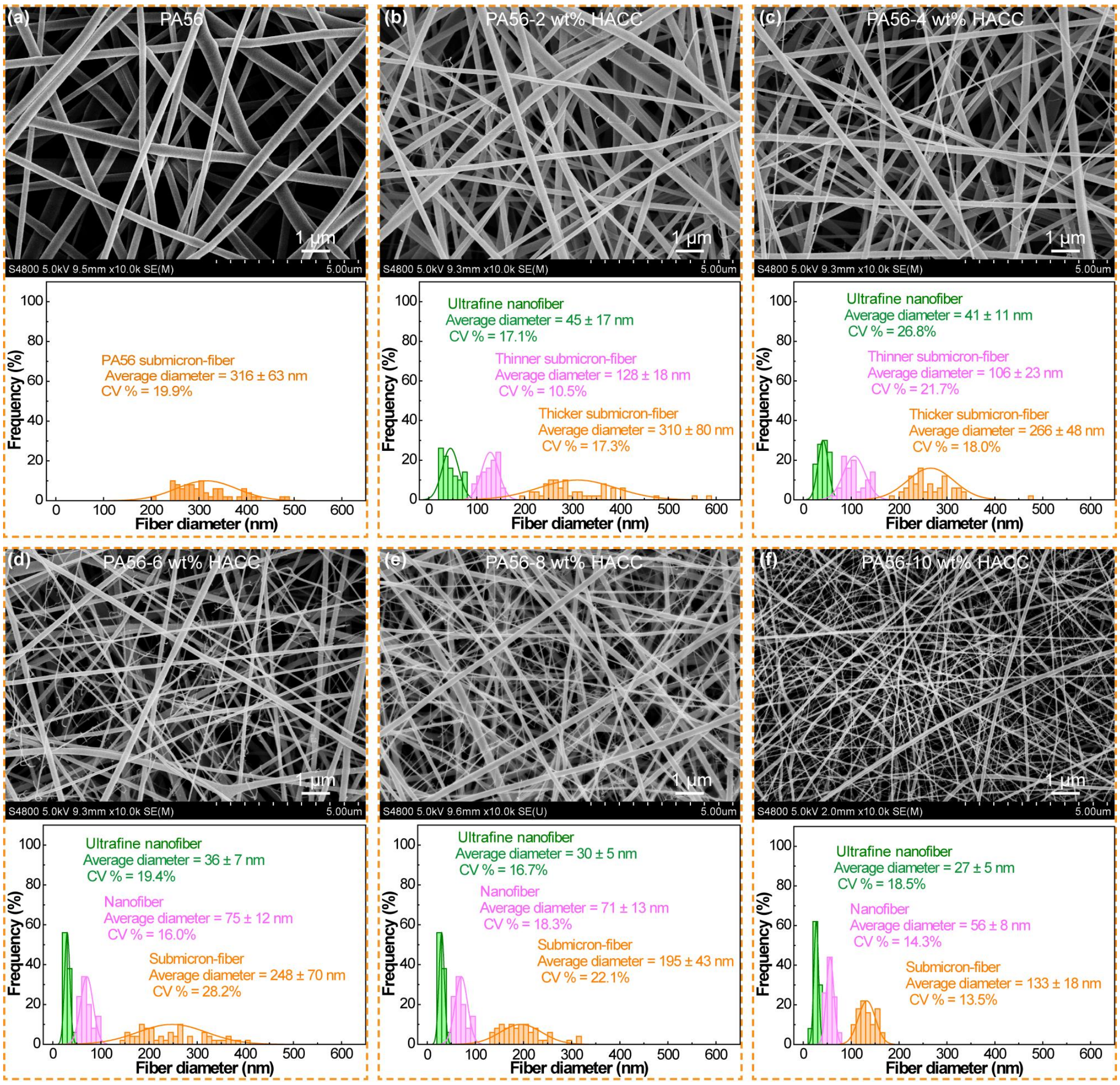

Fig. S5 Morphological characteristics of different PA56 fiber mats. (a) SEM images and fiber diameter distributions of mats prepared from 6 wt% PA56 solution. (b) SEM images and fiber diameter distributions of mats prepared from 6 wt% PA56/2 wt% HACC solution. (c) SEM images and fiber diameter distributions of mats prepared from 6 wt% PA56/4 wt% HACC solution. (d) SEM images and fiber diameter distributions of mats prepared from 6 wt% PA56/6 wt% HACC solution. (e) SEM images and fiber diameter distributions of mats prepared from 6 wt% PA56/8 wt% HACC solution. (f) SEM images and fiber diameter distributions of mats prepared from 6 wt% PA56/10 wt% HACC solution. Error bars represent the standard deviation of the measured properties and the samples are at least thirty. Source data are provided as a Source Data file.

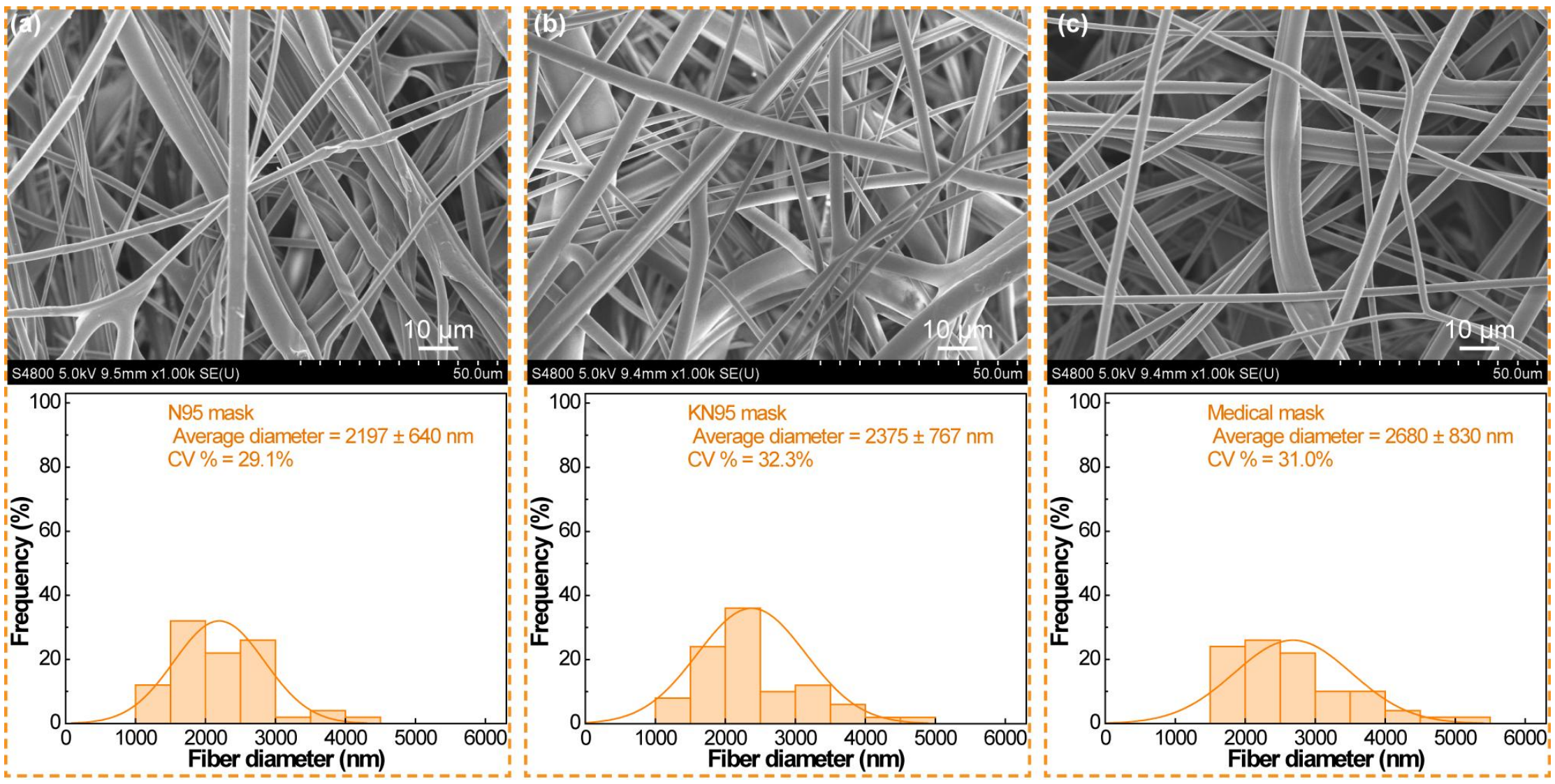

Fig. S6 Morphological characteristics of different commercial air filters. (a) SEM images and fiber diameter distributions of N95 mask. (b) SEM images and fiber diameter distributions of KN95 mask. (c) SEM images and fiber diameter distributions of medical mask. Error bars represent the standard deviation of the measured properties and the samples are at least fifty. Source data are provided as a Source Data file.

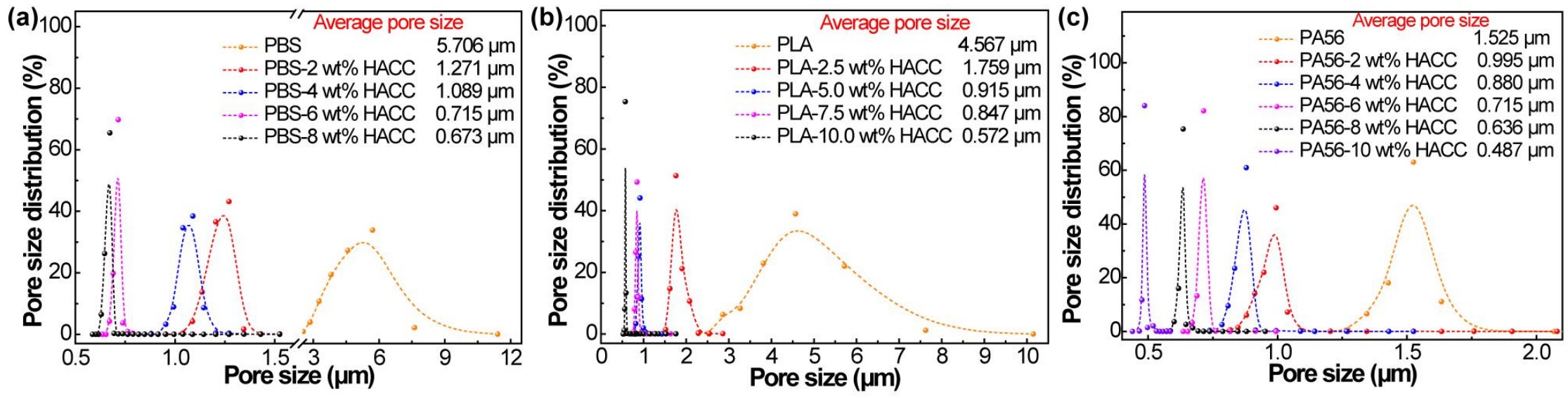

Fig. S7 Effect of HACC ratio on the pore structure of fiber membranes. (a) Pore size distribution of PBS fiber membranes prepared from PBS solutions with different HACC loadings. (b) Pore size distribution of PLA fiber membranes prepared from PBS solutions with different HACC loadings. (c) Pore size distribution of PA56 fiber membranes prepared from PA56 solutions with different HACC loadings. Source data are provided as a Source Data file.

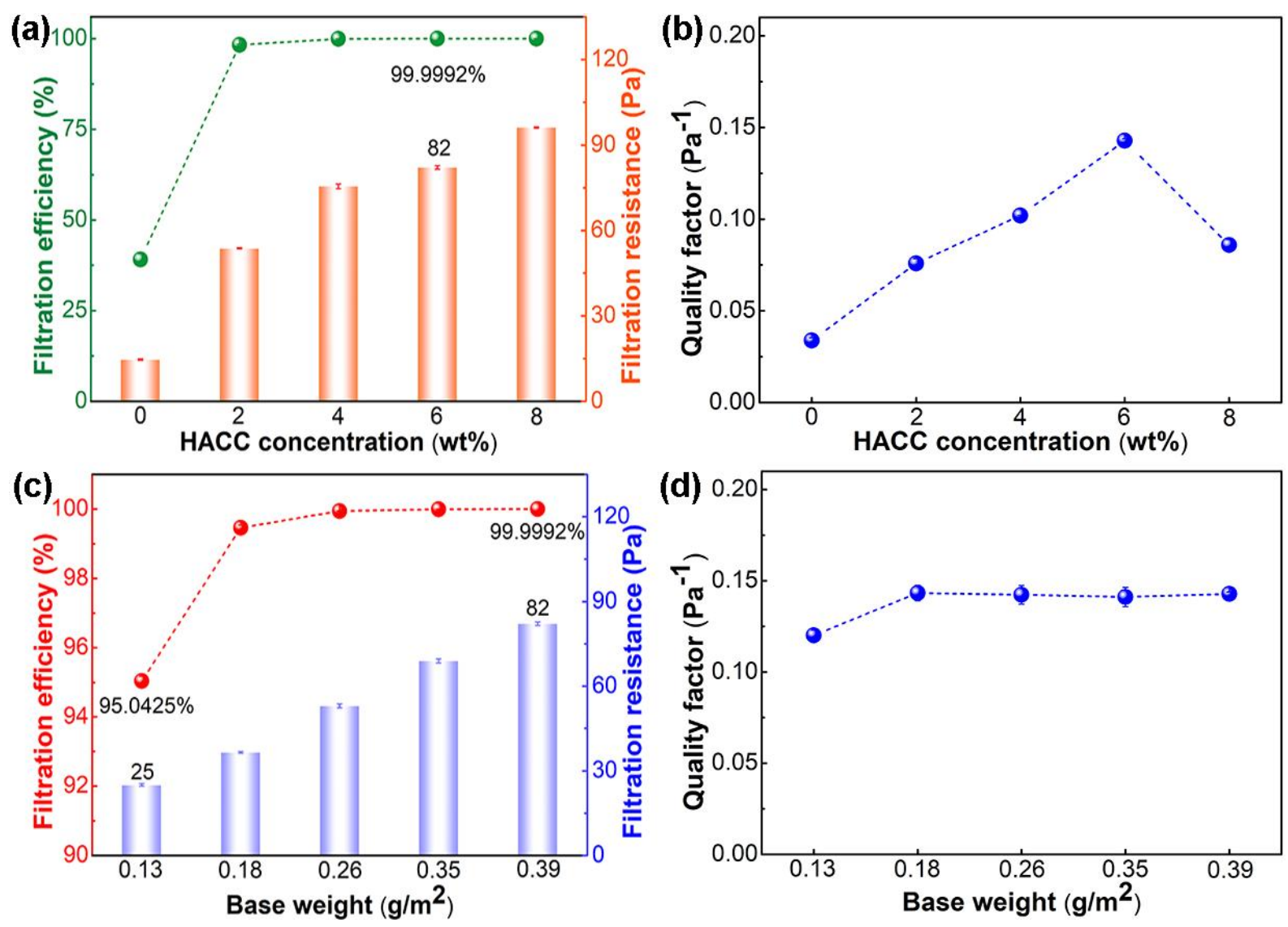

Fig. S8 Filtration performance optimization of PBS/HACC fiber membranes. (a) Filtration efficiency and resistance of PBS/HACC fiber mats prepared from PBS solutions doped with different HACC concentrations. (b) Quality factor of PBS/HACC fiber mats prepared from PBS solutions doped with different HACC concentrations. (c) Effect of base weight of PBS TSFM on its filtration efficiency and resistance. (d) Effect of base weight of PBS TSFM on its quality factor. Error bars represent the standard deviation of the measured properties and the samples are at least three. Source data are provided as a Source Data file.

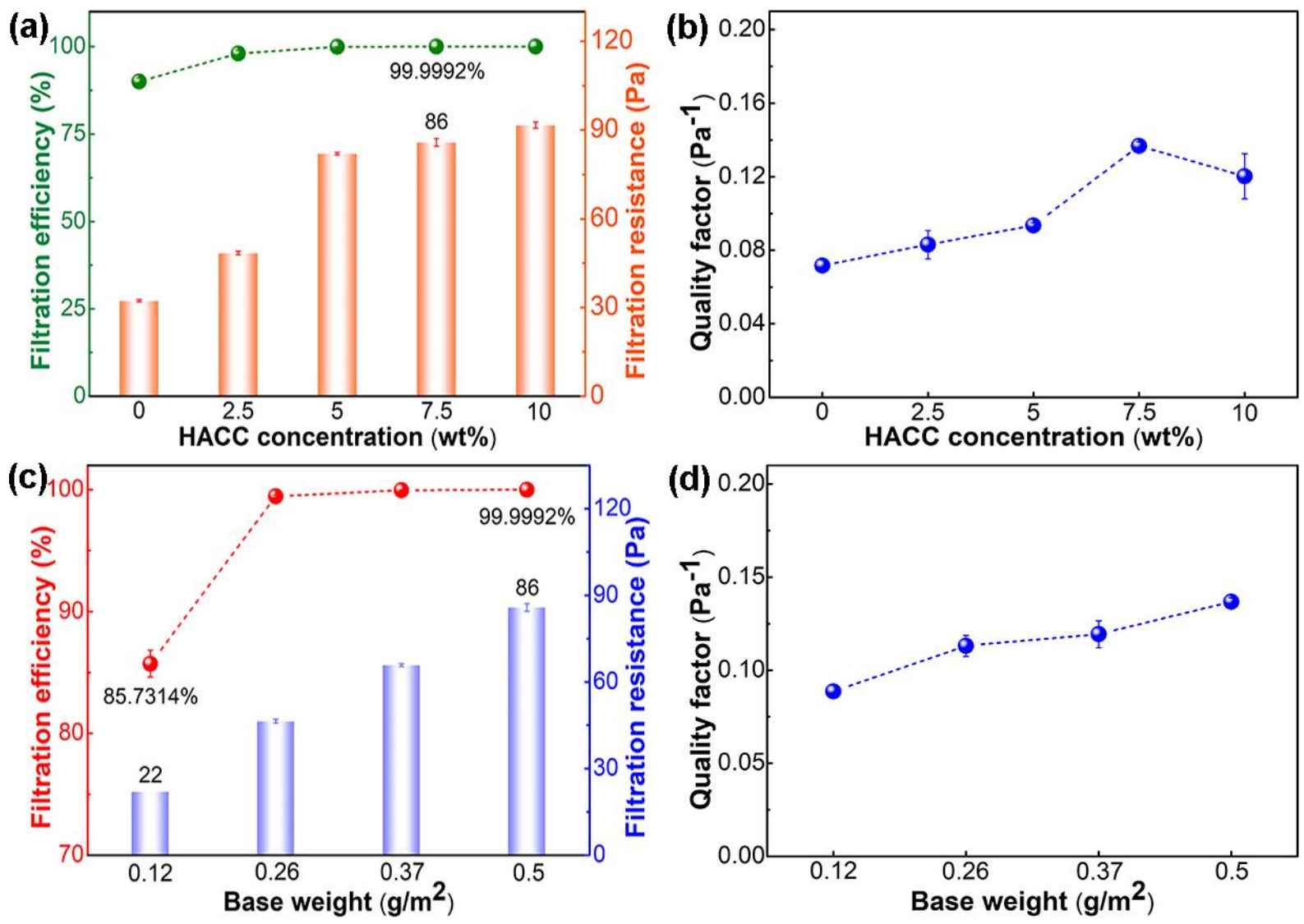

Fig. S9 Filtration performance optimization of PLA/HACC fiber membranes. (a) Filtration efficiency and resistance of PLA/HACC fiber mats prepared from PLA solutions doped with different HACC concentrations. (b) Quality factor of PLA/HACC fiber mats prepared from PLA solutions doped with different HACC concentrations. (c) Effect of base weight of PLA TSFM on its filtration efficiency and resistance. (d) Effect of base weight of PLA TSFM on its quality factor. Error bars represent the standard deviation of the measured properties and the samples are at least three. Source data are provided as a Source Data file.

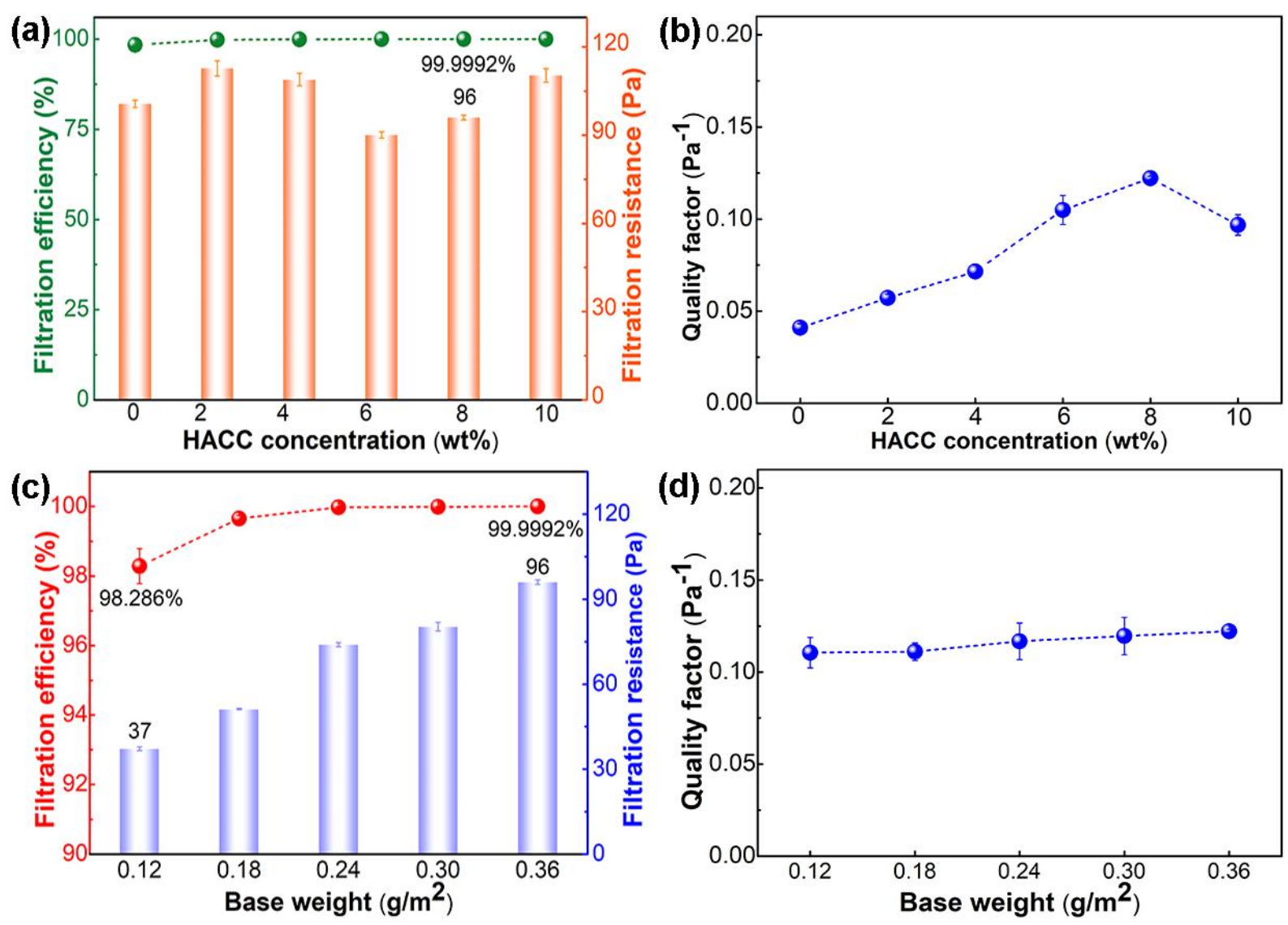

Fig. S10 Filtration performance optimization of PA56/HACC fiber membranes. (a) Filtration efficiency and resistance of PA56/HACC fiber mats prepared from PA56 solutions doped with different HACC concentrations. (b) Quality factor of PA56/HACC fiber mats prepared from PA56 solutions doped with different HACC concentrations. (c) Effect of base weight of PA56 TSFM on its filtration efficiency and resistance. (d) Effect of base weight of PA56 TSFM on its quality factor. Error bars represent the standard deviation of the measured properties and the samples are at least three. Source data are provided as a Source Data file.

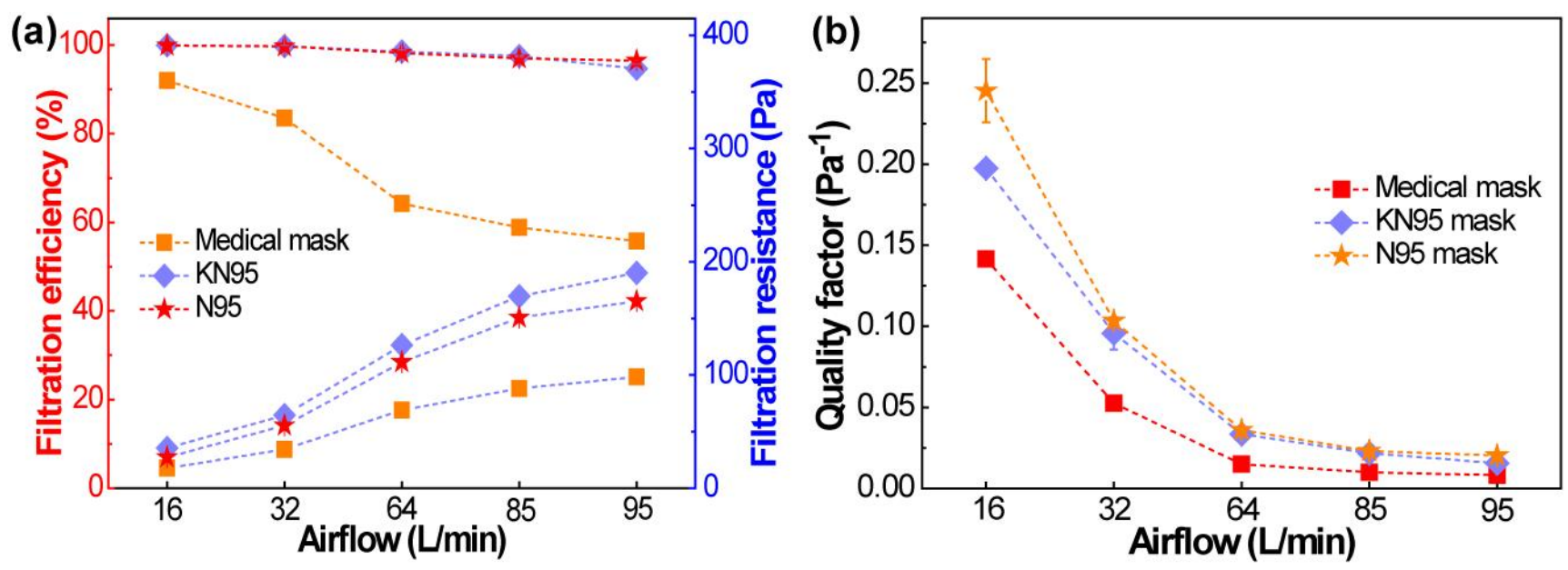

Fig. S11 Effect of airflow rate on filtration performance of commercial air filters. (a) Effect of airflow rate on filtration efficiency and resistance of medical masks, KN95 masks, and N95 masks. (b) Effect of airflow rate on quality factor of medical masks, KN95 masks, and N95 masks. Error bars represent the standard deviation of the measured properties and the samples are at least three. Source data are provided as a Source Data file.

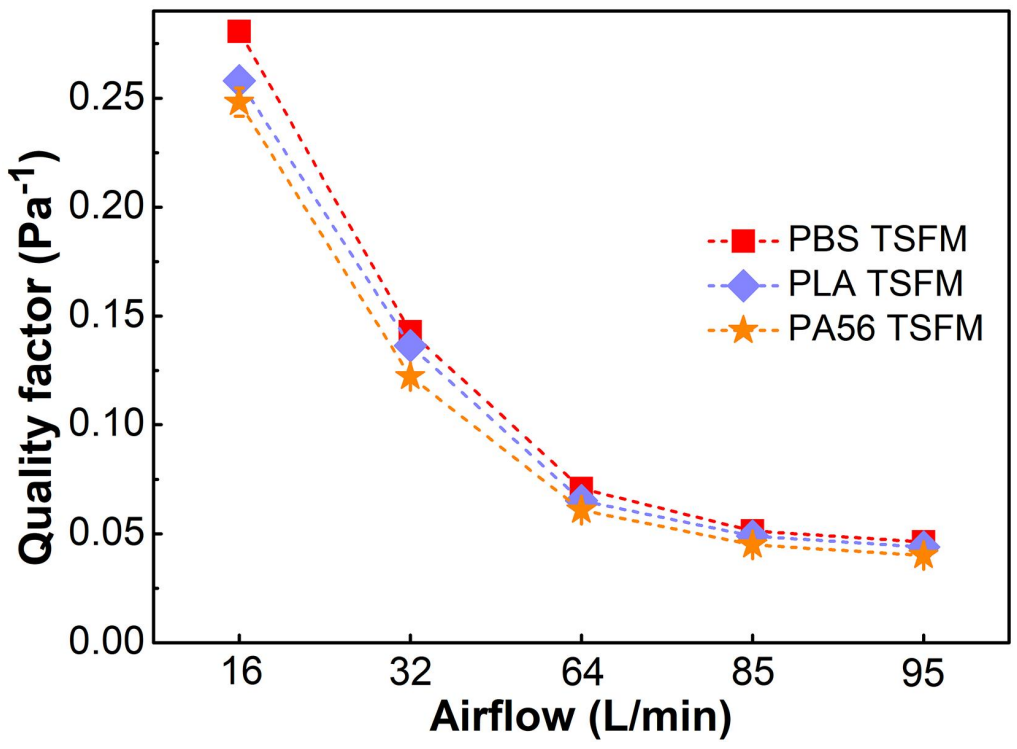

Fig. S12 Effect of airflow rate on qualify factor of PBS, PLA, PA56 TSFM. Error bars represent the standard deviation of the measured properties and the samples are at least three. Source data are provided as a Source Data file.

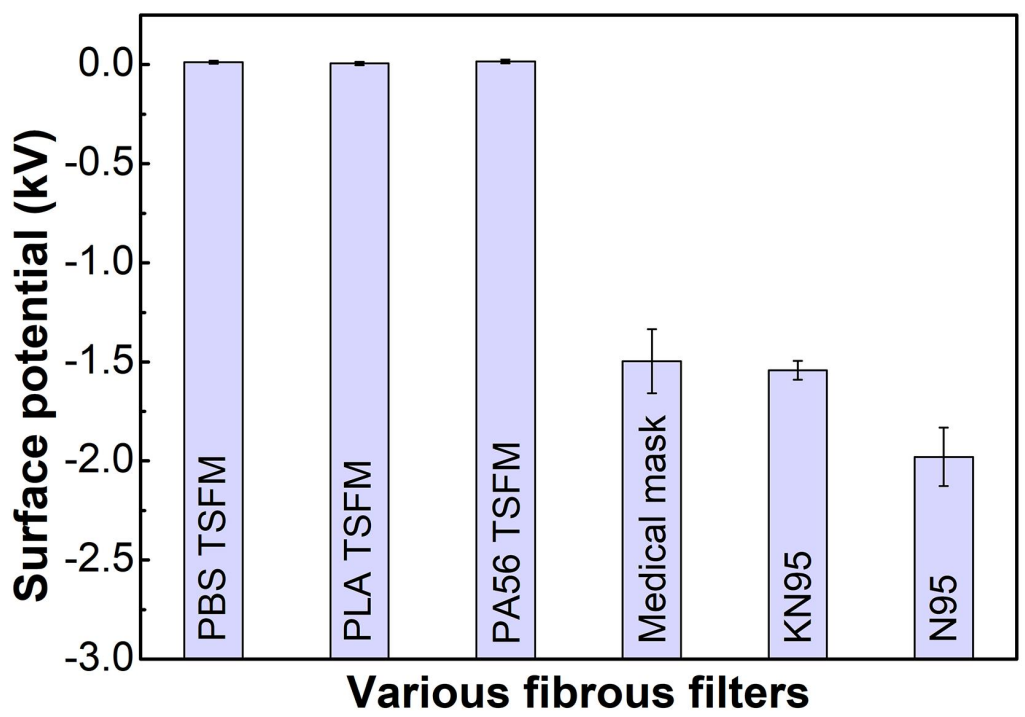

Fig. S13 Surface potential of various air filters. Error bars represent the standard deviation of the measured properties and the samples are at least three. Source data are provided as a Source Data file.

Table S1. Comparison of $PM_{0.3}$ filtration efficiency, filtration resistance, air permeability, and vapor permeability between this work and recent reports.

Raw materials	Base weight (g/m^2)	$PM_{0.3}$ filtration efficiency (%)	Filtration resistance (Pa)	QF (Pa^{-1})	Air permeability (mm/s)	Vapor permeability (g/m^2/day)	Reference
PBS/HACC (Biodegradable)	0.39	99.9992%	82	0.143	157	7400	This work
PLA/HACC (Biodegradable)	0.50	99.9992%	86	0.136	129	7302	This work
PA56/HACC (Biodegradable)	0.36	99.9992%	96	0.122	111	6818	This work
Glass fiber (Non-biodegradable)	-	99.9995%	319	0.038	-	-	1
Glass fiber (Non-biodegradable)	80	99.97%	234	0.035	-	-	2
Glass fiber/Si (Non-biodegradable)	70	97%	109	0.032	-	-	3
PP/Si (Non-biodegradable)	10	96%	67	0.048		-	4
PI/Ag (Non-biodegradable)	11.28	98%	42	0.093	37.5	-	5
PVDF/PTFE (Non-biodegradable)	5.4	96.1%	57	0.057	-	-	6
PA6/PVDF (Non-biodegradable)	6	98.1%	84	0.047	-	-	7
PA6/DTAC (Non-biodegradable)	0.57	99.95%	120	0.063	96	7541	8
PAN (Non-biodegradable)	0.6	99.73%	110	0.044	100	-	9
PAN (Non-biodegradable)	0.88	99.92%	89	0.080	80	-	10
PCL/LiCl (Biodegradable)	0.95	99.972%	125	0.065	-	-	11
PSU/SiO_2	1.45	99.8%	39	0.159	-	-	12

(Non-biodegradable)							
MSOA-PLA (Biodegradable)	29.6	99.12%	124	0.038	49.2	-	13
PA/PVDF (Non-biodegradable)	0.7	99.48%	34	0.155	-	-	14
Collagen (Biodegradable)	-	98%	70	0.056	-	-	15
Cellulose, Zein (Biodegradable)	10.2	99.99%	45	0.205	-	-	16
PVDF (Non-biodegradable)	0.75	99.995%	91	0.109	-	-	17
PLA (Biodegradable)	-	96.26%	40	0.082	-	5364	18

Table S2. Preparation parameters for electrospun fiber mats.

Samples	Polymer Concentration (wt%)	Mass ratio of HACC to polymer (wt%)	Voltage (kV)	Solution supply rate (mL/h)	Distance (cm)
PBS	10	0.0	7.5	3.0	12
PBS-2 wt%HACC	10	2.0	15	0.5	12
PBS-4 wt%HACC	10	4.0	15	0.5	12
PBS-6 wt%HACC	10	6.0	15	0.2	12
PBS-8 wt%HACC	10	8.0	25	0.3	12
PLA	8.0	0.0	8	3.0	12
PLA-2.5 wt%HACC	8.0	2.5	25	1.0	12
PLA-5.0 wt%HACC	8.0	5.0	20	0.5	12
PLA-7.5 wt%HACC	8.0	7.5	25	0.5	12
PLA-10 wt%HACC	8.0	10	20	0.5	12
PA56	6.0	0.0	10.5	1.5	12
PA56-2 wt%HACC	6.0	2.0	20	1.0	12
PA56-4 wt%HACC	6.0	4.0	20	0.8	12
PA56-6 wt%HACC	6.0	6.0	20	0.5	12
PA56-8 wt%HACC	6.0	8.0	20	0.3	12
PA56-10 wt%HACC	6.0	10	20	0.3	12

Supplementary Reference

1. Zhou ZQ*, et al.* Trichome-like biomimetic air filters via templated silicone nanofilaments. *Advanced Materials* 36, 2311129 (2024).
2. Zhou ZQ*, et al.* Conformal build-up of functionalized air filters with improved air cleaning and bioprotective traps. *Advanced Functional Materials* 34, 2306777 (2024).
3. Zhou ZQ*, et al.* Functionalized double-gradient janus filters for full-scale capture and inactivation of multiscale aqueous bioaerosols. *Advanced Functional Materials* 34, 2402969 (2024).
4. Zhou ZQ*, et al.* Bioinspired structures made of silicone nanofilaments for upcycling waste masks to reusable N95 respirators. *Nano Letters* 24, 4415-4422 (2024).
5. Lyu P*, et al.* Heat-resistant air filters based on self-sustained electrostatic and antibacterial polyimide/silver fiber mats. *Advanced Functional Materials* 34, 2400685 (2024).
6. Shi S*, et al.* An intelligent wearable filtration system for health management. *ACS Nano* 17, 7035-7046 (2023).
7. Peng ZH*, et al.* Self-charging electrostatic face masks leveraging triboelectrification for prolonged air filtration. *Nature Communications* 13, 7835 (2022).
8. Yang YC*, et al.* Ultrathin, ultralight dual-scale fibrous networks with high-infrared transmittance for high-performance, comfortable and sustainable $PM_{0.3}$ filter. *Nature Communications* 15, 1586 (2024).
9. Zhou MJ*, et al.* TriboNano shield-Scalable manufacturing anti-smog multi-level structured nanofiber air filter with co-enhanced purification performance towards self-powered window screen. *Nano Energy* 121, 109230 (2024).
10. Zhou MJ*, et al.* Ultralight electrospun composite filters with nertical ternary spatial network for high-performance $PM_{0.3}$ purification. *Advanced Materials* 37, 2419389 (2025).
11. Liu H*, et al.* High-performance integrated micro-vortex air filter enabled with honeycomb-like structured nanofibrous networks. *Advanced Materials* 37, 2419143 (2025).
12. Li XX*, et al.* An ultrathin membrane with bubble-raft-inspired single-layer nanofibrous networks for efficient and low-resistance air filtration. *Advanced Materials* 37, 2504379 (2025).
13. Wang CM*, et al.* Multiscale orientation armoring strategy to heat-resistant and high-selectivity nanofiber membranes for fireground mmergency. *ACS Nano* 20, 905-917 (2026).
14. Yang M*, et al.* Two-dimensional piezoelectric nanofibrous webs by self-polarized assembly for high-performance $PM_{0.3}$ filtration. *ACS Nano* 18, 16895-16904 (2024).
15. Wang JC*, et al.* Nanofibrillated collagen fiber networks for enhanced air purification. *Nature Communications* 16, 6823 (2025).

16. Wang QX*, et al.* Sustainable biomass-based filter for high-efficiency $PM_{0.3}$ filtration. *Nature Communications* 16, 6596 (2025).
17. Zhang SC, Liu H, Tang N, Zhou S, Yu JY, Ding B. Spider-web-inspired $PM_{0.3}$ filters based on self-sustained electrostatic nanostructured networks. *Advanced Materials* 32, 2002361 (2020).
18. Li XY, *et al.* Spider-web-inspired $PM_{0.3}$ filters based on self-sustained electrostatic nanostructured networks bioinspired multi-gradient structure enables environment-adaptive personal respiratory protection and moisture management for biodegradable poly(lactic acid) meta-membranes. *Advanced Functional Materials* e24202 (2025).
