# Supplementary material for: Sustainable trans-scale fibrous membranes for stable ultra-protective air filtration
Source: Nat Commun. 2026 Jun 23;17:7876. doi: 10.1038/s41467-026-74514-x (PMC13443542; doi:10.1038/s41467-026-74514-x)
Supplement: Supplementary file 2 [file 41467_2026_74514_MOESM2_ESM.pdf]

nature portfolio

Peer Review File

Sustainable trans-scale fibrous membranes for stable ultra-protective air filtration

Corresponding Author: Professor Yuekun Lai

This file contains all reviewer reports in order by version, followed by all author rebuttals in order by version.

Version 0:

Reviewer comments:

Reviewer #1

(Remarks to the Author)
In the paper entitled “Sustainable trans-scale fibrous membranes for stable ultra-protective air filtration”, the authors propose a universal multi-level jet splitting strategy for the one-step fabrication of trans-scale fiber membranes (TSFM) from various bio-based polymers. They exhibit stable filtration efficiencies of 99.999% for PM0.3 and 100% for PM0.5 and PM1.0. Furthermore, the ultra-protective TSFM offer superior wearing comfort. Notably, these TSFM are biodegradable and reduce raw polymer consumption by 99%, thus demonstrating exceptional environmental benignity and sustainability. The design concept presented in this work is novel and compelling. I recommend publication in Nature Communications after the authors address the following minor revisions.
1. “Therefore, the tailored supramolecular assembly induces the asymmetric multi-level jet splitting and enables the the fabrication of tran-scale fibers with diameter gradient.” What is the asymmetric multi-level jet splitting? A detailed clarification of the asymmetry should be provided.
2. “As presented in Fig. 3l, the pore size of PBS, PLA, PA56 SFM exceeded 1 μm, whereas the corresponding PBS, PLA, and PA56 TSFM showed significantly smaller pore size of 0.715, 0.847, and 0.636 μm, respectively.” Please provide a reasonable explanation for this observation. Some recent progress may be helpful to support the claims: ACS Appl. Mater. Interfaces 2026, 18 (4), 7440-7451; Chem. Eng. J. 2026, 530, 173106; 10.1002/adfm.202531069; ACS Nano 2026, 20 (1), 905-917; 10.1002/adfm.202524202; 10.1002/adfm.202525521.
3. “Notably, the overall tensile performance of PLA and PA56 TSFM surpassed that of commercial melt-blown nonwovens, while PBS TSFM demonstrated superior elongation at break with marginally lower tensile strength.” Does the mechanical property of PBS TSFM affect its practical application?
4. The abstract should be condensed to approximately 150 words as required by the journal.
5. Base weight measurements of the fibrous membranes should be supplemented in the Characterization.
6. The “Data availability” and “Author contributions” statements are omitted from the manuscript.

Reviewer #2

(Remarks to the Author)
The authors report a multi-level jet-splitting electrospinning strategy to fabricate hierarchical fibrous membranes for air filtration. The consideration of air permeability and heat/moisture transfer is valuable, which are important factors related to the wearing comfort of filtration materials. The manuscript contains a substantial amount of experimental data. However, several issues regarding the mechanism should be further clarified. I recommend for publication with minor revision pending the resolution of the following issues.
1. In Fig. 4c-d and Fig. S11, the filters exhibit stable filtration performance compared with commercial filtration media. Please clarify the mechanism responsible for this behavior.
2. Please clarify the reason for the slight increase in filtration resistance observed for PBS TSFM, PLA TSFM, and PA56 TSFM during the cyclic filtration process (Fig. 4g-i).
3. Can this multi-level jet splitting strategy be applied to other conventional spinning methods, such as melt spinning and solution spinning?
4. The authors attribute the high air permeability and rapid heat/moisture transfer to the ultrathin thickness (~1.2 μm) of the TSFM. However, the membrane is also described as having a dense submicron porous structure, which would generally reduce permeability. How does this filter maintain high air permeability despite the reported dense submicron porous

structure? Moreover, what structural features allow the TSFM to overcome the efficiency-permeability trade-off?
5. Fig. 6f, “based on a global daily demand of 2 billion masks”. What is the source of this estimation?
6. The sustainability discussion mainly relies on the ultralow basis weight of the TSFM filters, while the environmental benefits arising from material selection and real biodegradability are not sufficiently demonstrated.
7. The environmental degradability of PBS, PLA, and PA56 differs significantly. The authors claim the environmental compatibility of the TSFM filters, but the discussion does not address aspects required to support such claims, such as recycling strategies, filter reusability, or life-cycle assessment. Without considering these factors, the sustainability advantages remain insufficiently demonstrated, and the claim of environmental compatibility may be overstated.
8. Fig. 2 seems to miss the description for panel (g). Moreover, it is unclear how the jet-splitting process was directly observed using an optical microscope as described in Characterization, given the small scale and high velocity of electrospinning jets. Please clarify the experimental setup used for capturing these images.

Reviewer #3

(Remarks to the Author)
I co-reviewed this manuscript with one of the reviewers who provided the listed reports. This is part of the Nature Communications initiative to facilitate training in peer review and to provide appropriate recognition for Early Career Researchers who co-review manuscripts.

Reviewer #4

(Remarks to the Author)
This work proposes a universal multi-level splitting electrospinning strategy applicable to various bio-based polymers for fabricating ultra-protective, super-comfortable, and sustainable filtration media. The work exhibits remarkable innovation and practical application value. Nevertheless, there remain aspects that require enhancement in the presentation of specific content, data interpretation, mechanism analysis, and refinement of details. The specific revision suggestions are as follows:
1. Abstract. After stating "reducing polymer consumption by 99%", add a reference object (e.g., "compared with commercial N95 masks") to make the data more precise and avoid ambiguity for readers.
2. Introduction. When elaborating on the hazards of ultrafine aerosols, the specific manifestations of the technical bottlenecks of current commercial filter materials should be briefly supplemented so as to further highlight the necessity of this work.
3. Introduction. When elaborating on the interaction between HACC and bio-based polymers, it is advisable to supplement a schematic diagram of molecular action and briefly explain the specific principle of HFIP in reducing the surface tension of the solution.
4. The curves of conductivity, surface tension, and viscosity varying with the concentration of HACC in Figure 2 should be supplemented with an analysis of key data points in the main text (e.g., at what concentration of HACC the solution performance exhibits a turning - point and meets the optimal conditions for jet splitting), instead of simply describing the trends. Regarding the conclusion that "the interaction between the polymer and HACC hinders the migration of charged ions", electrochemical test data (such as ion mobility) can be added for verification to enhance the reliability of the mechanism.
5. The SEM image in Figure 2h can be utilized to add a statistical graph of fiber diameter distribution, which quantifies the diameter gradient of the cross-scale fibers.
6. The cross-sectional SEM images in Figures 3g-i should be labeled to provide more detailed explanations. The specific thickness values of the films need to be verified against the thickness data in Figure 5a. Regarding the conclusion that "excessive HACC addition reduces the solution spinnability", photos of fiber membrane defects (such as beads and broken filaments) at different HACC concentrations should be supplemented to visually illustrate the changes in spinnability.
7. When comparing the performance of TSFM with that of commercial masks at different air flow rates, the change curve of QF can be supplemented to more intuitively demonstrate the comprehensive filtration performance advantages of TSFM. For the cyclic filtration tests in Figure 4g-i, the microscopic morphology changes of the fiber membrane before and after filtration can be supplemented to analyze the structural reasons for its filtration stability.
8. Figure 6g, a brief degradation mechanism should be added.
9. Preparation of fiber membranes. During the electrospinning process, it is essential to supplement key process parameters, including the voltage of the high-voltage power supply, the receiving distance, the solution feeding rate, as well as the temperature and humidity of the spinning environment. A concise description of the structure of the "self-made electrospinning machine" can be provided, or a schematic diagram of the equipment can be added.
10. Please add the physical and chemical properties of the soil (e.g., pH, organic matter content, microbial community); clarify the initial state of the degradation samples (e.g., size, weight), and supplement the number of parallel experiments to ensure the reliability of the data.

Version 1:

Reviewer comments:

Reviewer #1

(Remarks to the Author)

The authors have made adequate revisions, and the revised version is ready for publication.

Reviewer #2

(Remarks to the Author)
All comments have been adequately addressed, and the manuscript is now suitable for publication.

Reviewer #3

(Remarks to the Author)
I co-reviewed this manuscript with one of the reviewers who provided the listed reports. This is part of the Nature Communications initiative to facilitate training in peer review and to provide appropriate recognition for Early Career Researchers who co-review manuscripts.

Reviewer #4

(Remarks to the Author)
Accept.

Open Access This Peer Review File is licensed under a Creative Commons Attribution 4.0 International License, which permits use, sharing, adaptation, distribution and reproduction in any medium or format, as long as you give appropriate credit to the original author(s) and the source, provide a link to the Creative Commons license, and indicate if changes were made.
In cases where reviewers are anonymous, credit should be given to 'Anonymous Referee' and the source.
The images or other third party material in this Peer Review File are included in the article's Creative Commons license, unless indicated otherwise in a credit line to the material. If material is not included in the article's Creative Commons license and your intended use is not permitted by statutory regulation or exceeds the permitted use, you will need to obtain permission directly from the copyright holder.
To view a copy of this license, visit https://creativecommons.org/licenses/by/4.0/

Dear editor and reviewers,

Thank you for your review and letter and for the reviewers' comments on our manuscript titled "***Sustainable trans-scale fibrous membranes for stable ultra-protective air filtration***" **(NCOMMS-26-016336-T)**. The comments are so wise and helpful for revising and improving our paper. After studying the comments carefully, we have revised our manuscript marked with a yellow background. Besides, the point-by-point answers to the reviewers' comments are listed as follows:

Reviewer #1 (Remarks to the Author):

In the paper entitled "Sustainable trans-scale fibrous membranes for stable ultra-protective air filtration", the authors propose a universal multi-level jet splitting strategy for the one-step fabrication of trans-scale fiber membranes (TSFM) from various bio-based polymers. They exhibit stable filtration efficiencies of 99.999% for $PM_{0.3}$ and 100% for $PM_{0.5}$ and $PM_{1.0}$. Furthermore, the ultra-protective TSFM offer superior wearing comfort. Notably, these TSFM are biodegradable and reduce raw polymer consumption by 99%, thus demonstrating exceptional environmental benignity and sustainability. The design concept presented in this work is novel and compelling. I recommend publication in Nature Communications after the authors address the following minor revisions.

Answer: We appreciate your affirmation of our research and valuable comments. We have carefully revised our manuscript point-by-point according to the comments.

Q1: "Therefore, the tailored supramolecular assembly induces the asymmetric multi-level jet splitting and enables the fabrication of trans-scale fibers with diameter gradient." What is the asymmetric multi-level jet splitting? A detailed clarification of the asymmetry should be provided.

A1: Thank you for the helpful suggestion. As shown in Fig. 2g, during the electrospinning process, multiple secondary jets and fine jets are generated from the main jet and secondary jets, respectively. Therefore, the asymmetric multi-level jet splitting describes the process whereby a primary initial jet transforms into numerous secondary and fine jets that exhibit a diameter gradient. The above detailed clarification has been added to the revised manuscript.

Q2: "As presented in Fig. 3l, the pore size of PBS, PLA, PA56 SFM exceeded 1 μm, whereas the corresponding PBS, PLA, and PA56 TSFM showed significantly smaller pore size of 0.715, 0.847, and 0.636 μm, respectively." Please provide a reasonable

explanation for this observation. Some recent progress may be helpful to support the claims: ACS Appl. Mater. Interfaces 2026, 18 (4), 7440-7451; Chem. Eng. J. 2026, 530, 173106; 10.1002/adfm.202531069; ACS Nano 2026, 20 (1), 905-917; 10.1002/adfm.202524202; 10.1002/adfm.202525521.

A2: Thanks for your kind comment. The pore size of the membrane is positively correlated with the fiber diameter. As presented in Fig. 3l, PBS, PLA, and PA56 SFM comprising 200-1000 nm submicron fibers possessed pore sizes larger than 1 μm. In contrast, PBS, PLA, and PA56 TSFM were composed of ultrafine nanofibers, nanofibers, and submicron fibers with considerably finer diameters, exhibiting remarkably smaller pore sizes of 0.715, 0.847, and 0.636 μm, respectively. Related explanations have been supplemented in the revised manuscript.

Q3: "Notably, the overall tensile performance of PLA and PA56 TSFM surpassed that of commercial melt-blown nonwovens, while PBS TSFM demonstrated superior elongation at break with marginally lower tensile strength." Does the mechanical property of PBS TSFM affect its practical application?

A3: Thanks a lot for your comment. As shown in Fig. 3o, the tensile stress of PBS TSFM is significantly enhanced when compared to PBS SFM and is close to that of melt-blown nonwovens. Besides, the commercial spunbond nonwovens are utilized as the mechanical support for PBS TSFM during its practical application. Fig. 4c, 4d, and 4g also demonstrate that PBS TSFM can meet the mechanical requirements of practical applications.

Q4: The abstract should be condensed to approximately 150 words as required by the journal.

A4: Thanks a lot for your kind suggestions. In accordance with the journal's requirements, the abstract has been condensed to within 150 words.

Q5: Base weight measurements of the fibrous membranes should be supplemented in the Characterization.

A5: Thank you for your kind reminder. Base weight measurements of the fibrous membranes have been supplemented in the Characterization.

Q6: The "Data availability" and "Author contributions" statements are omitted from the manuscript.

A6: Thanks a lot for your kind suggestions. The "Data availability", "Author contributions", and "Competing interests" have been added in the revised manuscript.

Reviewer #2 (Remarks to the Author):

The authors report a multi-level jet-splitting electrospinning strategy to fabricate hierarchical fibrous membranes for air filtration. The consideration of air permeability and heat/moisture transfer is valuable, which are important factors related to the wearing comfort of filtration materials. The manuscript contains a substantial amount of experimental data. However, several issues regarding the mechanism should be further clarified. I recommend for publication with minor revision pending the resolution of the following issues.

Answer: We sincerely appreciate your thorough review and insightful comments. We are truly grateful for your professional guidance, which has greatly helped us improve the quality of this manuscript. We have carefully taken all suggestions into account and extensively revised the manuscript to fully address the concerns you have raised.

Q1: In Fig. 4c-d and Fig. S11, the filters exhibit stable filtration performance compared with commercial filtration media. Please clarify the mechanism responsible for this behavior.

A1: Thanks a lot for your comment. In contrast to the surface potential of commercial electret melt-blown fibers (> -1 kV), that of TSFM was close to 0 V (Fig. S13). This result strongly indicates that commercial filters primarily capture aerosols via electrostatic attraction, whereas TSFM relies on a non-electrostatic filtration mechanism. Moreover, high airflow rates shorten the residence time of particulate matter within air filters, thereby impairing both physical sieving and electrostatic interception of commercial filters. This fundamental difference accounts for the unstable PM capture efficiency of commercial filters and the sustained filtration performance of TSFM filters[1-4]. The above explanations have been added to the revised manuscript.

[1] Zhou ZQ, et al. Trichome-like biomimetic air filters via templated silicone nanofilaments. *Advanced Materials* 36, 2311129 (2024).

[2] Zheng GF, et al. Green electrospinning fully bio-based lightweight nanofibrous membrane for high-performance and antibacterial air filtration via small molecule mutual support mechanism. *Journal of Cleaner Production* 486, 144562 (2025).

[3] Li XX, et al. An ultrathin membrane with bubble-raft-inspired single-layer nanofibrous networks for efficient and low-resistance air filtration. *Advanced Materials* 37, 2504379 (2025).
[4] Yang YC, et al. Ultrathin, ultralight dual-scale fibrous networks with high-infrared transmittance for high-performance, comfortable and sustainable $PM_{0.3}$ filter. *Nature Communications* 15, 1586 (2024).

Q2: Please clarify the reason for the slight increase in filtration resistance observed for PBS TSFM, PLA TSFM, and PA56 TSFM during the cyclic filtration process (Fig. 4g-i).

A2: Thank you for the valuable question. During cyclic filtration, the gradual deposition of particulate matter within the filter media partly blocks its pores and narrows the airflow channels[1-3]. This results in a slight increase in filtration resistance of three TSFM filters. Explanations have been added in the revised manuscript. The above explanations have been supplemented in the revised manuscript.

[1] Yang YC, et al. Sandwich-structured bimodal fiber/bead-on-string fiber composite membrane for comfortable $PM_{0.3}$ filter. *Chemical Engineering Journal* 495, 153245 (2024).
[2] Yang YC, et al. Ultrathin, ultralight dual-scale fibrous networks with high-infrared transmittance for high-performance, comfortable and sustainable $PM_{0.3}$ filter. *Nature Communications* 15, 1586 (2024).
[3] Zhou MJ, et al. Ultralight electrospun composite filters with nertical ternary spatial network for high-performance $PM_{0.3}$ purification. *Advanced Materials* 37, 2419389 (2025).

Q3: Can this multi-level jet splitting strategy be applied to other conventional spinning methods, such as melt spinning and solution spinning?

A2: Thanks for your insightful question. In this work, the synergistic effect of polyelectrolyte and high-voltage electrostatic field facilitates multi-level jet splitting. By contrast, melt spinning and solution spinning refine polymer fibers via mechanical roller drafting. Thus, the multi-level jet splitting strategy cannot be directly extended to other conventional spinning methods. Nevertheless, it still offers a valuable reference. Our research group is dedicated to developing multi-level jet splitting technologies for melt spinning and solution spinning by exploring innovative

fundamental mechanisms.

<u>Q4:</u> The authors attribute the high air permeability and rapid heat/moisture transfer to the ultrathin thickness (~1.2 μm) of the TSFM. However, the membrane is also described as having a dense submicron porous structure, which would generally reduce permeability. How does this filter maintain high air permeability despite the reported dense submicron porous structure? Moreover, what structural features allow the TSFM to overcome the efficiency-permeability trade-off?

<u>A4:</u> The high air permeability is primarily attributed to the ultrathin thickness (≈1.2 μm) and the hierarchical stacking architecture of trans-scale fibers within the TSFM. The ultrathin thickness of TSFM effectively shortens the gas transport path, and the hierarchical stacking architecture of trans-scale fibers provides abundant and interconnected transfer channels, which contribute to the superior air permeability.

The hierarchical stacking architecture of ultrafine trans-scale fibers endows the filtration media with ultrathin thickness, ultralow pore diameters, a high specific surface area, curved multi-level interception networks, and abundant interconnected transfer channels. These structural features enable the TSFM to overcome the trade-off between filtration efficiency and air permeability.

<u>Q5:</u> Fig. 6f, “based on a global daily demand of 2 billion masks”. What is the source of this estimation?

<u>A5:</u> Thanks for the comment. The estimate is derived from global daily demand data throughout the COVID-19 pandemic [1]. Relevant explanations and references have been added in the revised manuscript.

[1] Yang YC, et al. Ultrathin, ultralight dual-scale fibrous networks with high-infrared transmittance for high-performance, comfortable and sustainable $PM_{0.3}$ filter. *Nature Communications* 15, 1586 (2024).

<u>Q6:</u> The sustainability discussion mainly relies on the ultralow basis weight of the TSFM filters, while the environmental benefits arising from material selection and real biodegradability are not sufficiently demonstrated.

<u>A6:</u> Thanks a lot for your meaningful comment. The sustainability of air filters encompasses two core aspects of structural design and material selection: (i) The filters feature an ultralow basis weight, which significantly reduces raw material consumption; (ii) The filters are fabricated from biodegradable polymers, thus

exhibiting excellent environmental friendliness. Commercial air filter products (e.g., N95 masks) are manufactured from non-biodegradable petroleum-based polypropylene (PP) and are discarded in large quantities after single use. Direct incineration of these discarded masks inevitably causes severe air pollution, while landfilling them inflicts lasting damage to the ecological environment. Furthermore, disposable PP masks can degrade into microplastics, which pose a threat to microbial survival and human health through bioaccumulation and food chain transmission. These biodegradable TSFM filters can effectively avoid the post-use recycling and offer a secure, economical end-of-life disposal solution aligned with international sustainable development benchmarks[1-3]. The above explanations have been supplemented in the revised manuscript.

[1] Zhang LS, Leung MY, Boriskina S, Tao XM. Advancing life cycle sustainability of textiles through technological innovations. *Nature Sustainability* 6, 243-253 (2023).

[2] Wang JC, et al. Nanofibrillated collagen fiber networks for enhanced air purification. *Nature Communications* 16, 6823 (2025).

[3] Wang QX, et al. Sustainable biomass-based filter for high-efficiency $PM_{0.3}$ filtration. *Nature Communications* 16, 6596 (2025).

Q7: The environmental degradability of PBS, PLA, and PA56 differs significantly. The authors claim the environmental compatibility of the TSFM filters, but the discussion does not address aspects required to support such claims, such as recycling strategies, filter reusability, or life-cycle assessment. Without considering these factors, the sustainability advantages remain insufficiently demonstrated, and the claim of environmental compatibility may be overstated.

A7: Thanks a lot for your kind comment. We fully agree that PBS, PLA, and PA56 exhibit distinct environmental degradability characteristics. We have clarified that TSFM filters are designed as disposable biodegradable air filters; their sustainability is achieved through complete degradation in the natural environment rather than recycling or reuse, which is more in line with the actual application scenarios of disposable air filtration materials. The above explanations have been added to the revised manuscript.

Q8: Fig. 2 seems to miss the description for panel (g). Moreover, it is unclear how the jet-splitting process was directly observed using an optical microscope as described in Characterization, given the small scale and high velocity of electrospinning jets.

Please clarify the experimental setup used for capturing these images.

A8: Thank you very much for the valuable suggestions. The description for panel (g) has been supplemented in the revised manuscript. A segment of the split jet is intercepted by a rapidly moving glass slide oriented perpendicular to the direction of jet motion, and the morphology of the split jet is observed under an optical microscope. The above details have been added to the Characterization.

Reviewer #3 (Remarks to the Author):

I co-reviewed this manuscript with one of the reviewers who provided the listed reports. This is part of the Nature Communications initiative to facilitate training in peer review and to provide appropriate recognition for Early Career Researchers who co-review manuscripts.

Answer: We greatly appreciate your time and efforts as a co-reviewer of this manuscript, as well as your valuable contributions to the peer review process under the Nature Communications early-career researcher co-review initiative. We have carefully considered and thoroughly addressed all comments provided in the review report, and we have revised the manuscript accordingly to improve its quality and rigor. Thank you again for your constructive feedback and support.

Reviewer #4 (Remarks to the Author):

This work proposes a universal multi-level splitting electrospinning strategy applicable to various bio-based polymers for fabricating ultra-protective, super-comfortable, and sustainable filtration media. The work exhibits remarkable innovation and practical application value. Nevertheless, there remain aspects that require enhancement in the presentation of specific content, data interpretation, mechanism analysis, and refinement of details. The specific revision suggestions are as follows:

Answer: We sincerely appreciate the reviewer for highly recognizing the innovation, universal applicability, and practical value of our proposed multi-level splitting electrospinning strategy for bio-based polymers in constructing ultra-protective, super-comfortable, and sustainable filtration media. We are grateful for the constructive and detailed suggestions that are crucial for improving the quality of this manuscript.

Q1: Abstract. After stating "reducing polymer consumption by 99%", add a reference

object (e.g., "compared with commercial N95 masks") to make the data more precise and avoid ambiguity for readers.

A1: We sincerely appreciate your constructive suggestion. Following the comment, the description has been revised to “reducing polymer consumption by 99% compared with commercial N95 masks”.

Q2: Introduction. When elaborating on the hazards of ultrafine aerosols, the specific manifestations of the technical bottlenecks of current commercial filter materials should be briefly supplemented so as to further highlight the necessity of this work.

A2: Thank you for the valuable suggestion. possess the characteristics of micro-scale pores, high areal density (>50 g/ m^{-2}) and considerable thickness (>100 μm)[1-3]. Nevertheless, they suffer from insufficient protective capacity below 99% to the most permeable $PM_{0.3}$[1-2]. The bulky filter structure severely impedes the transfer of airflow, moisture, and heat, which significantly compromises wearing comfort[4]. Besides, disposable air filters with high base weight consume large quantities of raw materials, and they even led to acute supply shortages during the epidemic period[5-6]. Meanwhile, the massive discard of non-degradable filters in that period also generated over 8 million tons of persistent plastic waste, which in turn endangers terrestrial ecosystems, marine environments, and living organisms[5-7]. Consequently, the development of advanced air filters that can simultaneously improve protective performance, wearing comfort, and sustainability is urgently required. The above manifestations of technical bottlenecks in current commercial filter materials have been added in the revised manuscript.

[1] Park J, *et al.* Bioinspired capillary force-driven super-adhesive filter. *Nature* 643, 388-394 (2025).

[2] Zhou ZQ, *et al.* Functionalized double-gradient janus filters for full-scale capture and inactivation of multiscale aqueous bioaerosols. *Advanced Functional Materials* 34, 2402969 (2024).

[3] You J, *et al.* PPS gradient filter material with high efficiency and excellent dust removal capacity for industrial flue gas treatment by integrating numerical modeling and melt-blown-lamination technology. *Environmental Science & Technology* 59, 10368-10379 (2025).

[4] Yang YC, *et al.* Sandwich-structured bimodal fiber/bead-on-string fiber composite membrane for comfortable $PM_{0.3}$ filter. *Chemical Engineering Journal* 495, 153245 (2024).

[5] Peng YM, Wu PP, Schartup AT, Zhang YX. Plastic waste release caused by COVID-19 and its fate in the global ocean. *Proceedings of the National Academy of Sciences of the United States of America* 118, e2111530118 (2021).

[6] Woodward J, Li JW, Rothwell J, Hurley R. Acute riverine microplastic

contamination due to avoidable releases of untreated wastewater. *Nature Sustainability* 4, 793-802 (2021).
[7] Zhang LS, Leung MY, Boriskina S, Tao XM. Advancing life cycle sustainability of textiles through technological innovations. *Nature Sustainability* 6, 243-253 (2023).

Q3: Introduction. When elaborating on the interaction between HACC and bio-based polymers, it is advisable to supplement a schematic diagram of molecular action and briefly explain the specific principle of HFIP in reducing the surface tension of the solution.

A3: We appreciate your kind suggestions. A schematic diagram of intermolecular interactions between HACC and bio-based polymers is shown in Fig. 1a. HFIP lowers the surface tension of the spinning solution owing to its distinctive molecular composition and structure. The two bulky trifluoromethyl groups ($-CF_3$) in HFIP possess low polarizability and strong steric hindrance, which drastically weaken intermolecular forces and cohesive forces between liquid molecules. The above illustrations have been supplemented in the revised text.

[1] David Leboeuf. The rise and future of hexafluoroisopropanol as a solvent. *Trends in Chemistry* 7, 205-207 (2025).

Q4: The curves of conductivity, surface tension, and viscosity varying with the concentration of HACC in Figure 2 should be supplemented with an analysis of key data points in the main text (e.g., at what concentration of HACC the solution performance exhibits a turning - point and meets the optimal conditions for jet splitting), instead of simply describing the trends. Regarding the conclusion that "the interaction between the polymer and HACC hinders the migration of charged ions", electrochemical test data (such as ion mobility) can be added for verification to enhance the reliability of the mechanism.

A4: Thanks a lot for your professional and constructive comments. In the revised manuscript, we have supplemented a detailed analysis of key data points corresponding to the conductivity, surface tension, and viscosity curves as functions of HACC concentration in Figure 2. Specifically, we have identified and discussed the optimal conditions for jet splitting. As depicted in Fig. 3d-f, three types of uniform TSFM were fabricated from 10 wt%PBS/6 wt%HACC, 8 wt%PLA/7.5 wt%HACC, and 6 wt%PA56/8 wt%HACC solutions, respectively.

As shown in Fig. S2, the conductivity of PBS/HFIP, PLA/HFIP, and PA56/HFIP

solutions was nearly zero, whereas HACC/HFIP solutions with different ratios exhibited the highest conductivity. Notably, the conductivity of ternary solutions containing polymer, HACC, and HFIP was significantly lower than that of binary HACC-HFIP solutions. It demonstrated that the introduction of polymers such as PBS, PLA, and PA56 significantly decreased the solution conductivity. This result indicates that polymer-HACC interactions hinder the migration of charged ions. Relevant explanations have been improved in the updated manuscript.

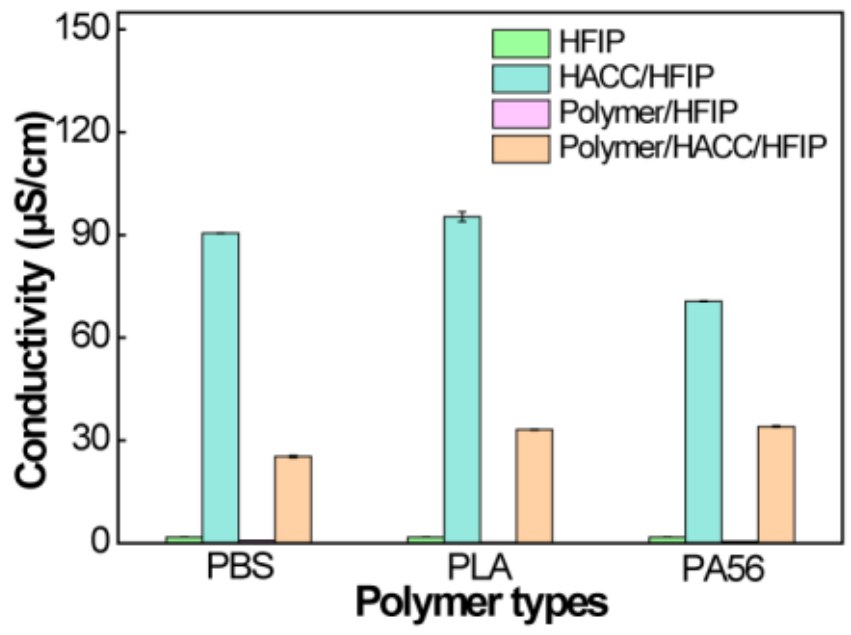

Figure. Conductivity of HFIP, HACC/HFIP, polymer/HFIP, and polymer/HACC/HFIP solutions.

Q5: The SEM image in Figure 2h can be utilized to add a statistical graph of fiber diameter distribution, which quantifies the diameter gradient of the cross-scale fibers.

A5: Thank you for the kind suggestion. The diameter distribution of trans-scale fibers is provided in Fig. 3k and Fig. S3-S5, which quantifies the diameter gradient of trans-scale fibers.

Q6: The cross-sectional SEM images in Figures 3g-i should be labeled to provide more detailed explanations. The specific thickness values of the films need to be verified against the thickness data in Figure 5a. Regarding the conclusion that "excessive HACC addition reduces the solution spinnability", photos of fiber membrane defects (such as beads and broken filaments) at different HACC concentrations should be supplemented to visually illustrate the changes in spinnability.

A6: We sincerely appreciate your kind suggestions. Fig. 3g-i has been improved, and more details have also been added in the revised manuscript. The specific thickness values of the membranes in Fig. 3g-i are verified by the thickness data in Figure 5a. As shown in the figure below and Fig. S3-S5, excessive HACC addition reduces the solution spinnability and thus causes membrane defects such as broken fibers.

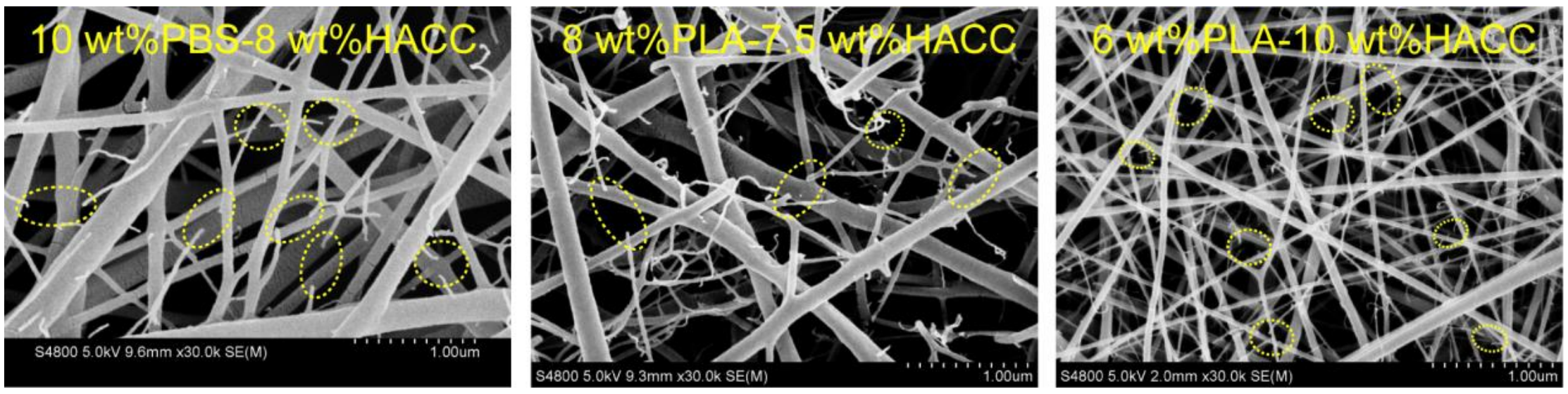

Figure. SEM images of three kinds of membranes with broken fibers.

<u>Q7:</u> When comparing the performance of TSFM with that of commercial masks at different air flow rates, the change curve of QF can be supplemented to more intuitively demonstrate the comprehensive filtration performance advantages of TSFM. For the cyclic filtration tests in Figure 4g-i, the microscopic morphology changes of the fiber membrane before and after filtration can be supplemented to analyze the structural reasons for its filtration stability.

<u>A7:</u> We sincerely appreciate your constructive and detailed suggestions. We have supplemented the quality factor (QF) curves of TSFM and commercial masks at different airflow rates in the revised manuscript. As shown below, TSFM showed a higher quality factor than that of three kinds of commercial masks under different airflow rates, demonstrating the superior comprehensive filtration performance of TSFM.

The microscopic morphology of TSFM before filtration and after cyclic filtration is presented in Fig. 3d-f and Fig. 4j, respectively. Through comparative observation of the fiber morphology, we have further elaborated on the excellent structural integrity and dimensional stability of the membrane, which directly reveals the structural mechanism responsible for its stable filtration performance during cyclic testing.

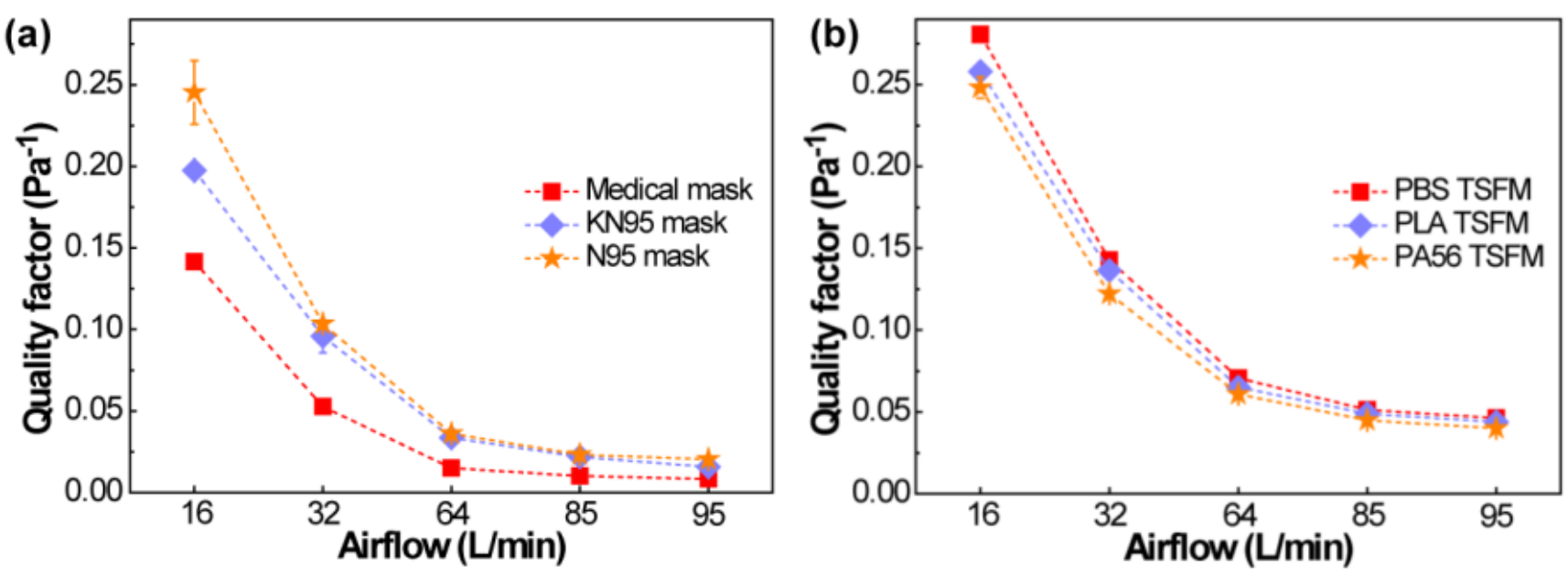

Figure. Effect of airflow rate on qualify factor of (a) commercial masks, and (b) TSFM.

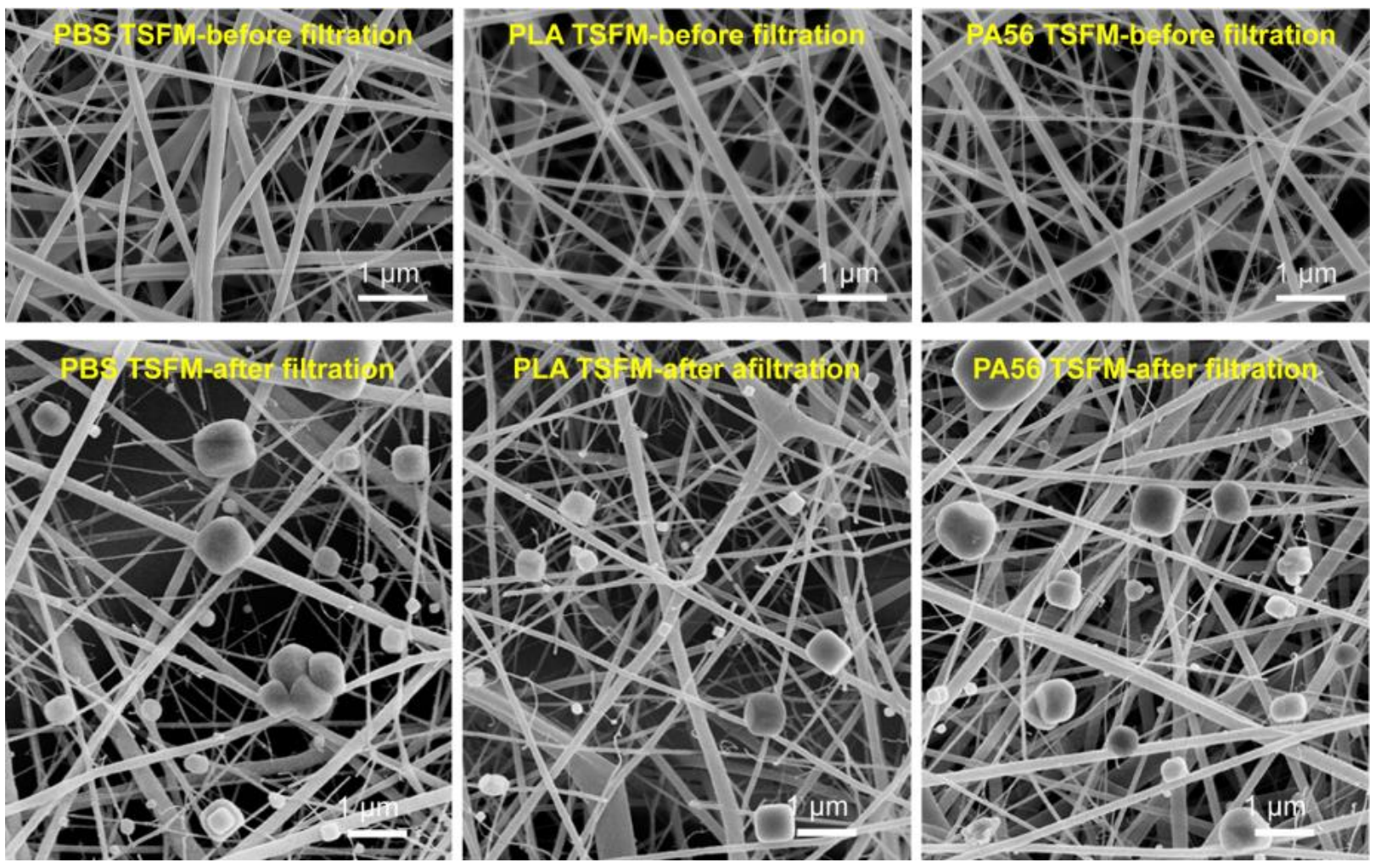

Figure. SEM images of TSFM filters before and after filtration.

Q8: Figure 6g, a brief degradation mechanism should be added.

A8: Thanks a lot for your insightful advice. These biopolymers can undergo enzymatic hydrolysis and microbial degradation, and ultimately decompose into CO_2 and H_2O under warm and humid natural environmental conditions[1-2]. The above explanations have been added to the revised manuscript.

[1] Rajgond, V., Mohite, A., More, N. et al. Biodegradable polyester-polybutylene succinate (PBS): a review. *Polymer Bulletin* 81, 5703-5752 (2024).

[2] Wang, CM, *et al*. Spider silk-inspired spinning of physically crosslinked ultrafine and electroactive poly(lactic acid) nanofibers for high-humidity PM filtration and intelligent respiratory diagnosis. *Chemical Engineering Journal* 530, 173106 (2026).

Q9: Preparation of fiber membranes. During the electrospinning process, it is essential to supplement key process parameters, including the voltage of the high-voltage power supply, the receiving distance, the solution feeding rate, as well as the temperature and humidity of the spinning environment. A concise description of the structure of the "self-made electrospinning machine" can be provided, or a schematic diagram of the equipment can be added.

A9: Thank you for the valuable comments. Key process parameters, including the voltage of the high-voltage power supply, the receiving distance, and the solution feeding rate, have been provided in Table S2. The temperature and humidity of the spinning environment were added in the Methods. The detailed information about the electrospinning machine has also been added to the revised manuscript.

<u>Q10:</u> Please add the physical and chemical properties of the soil (e.g., pH, organic matter content, microbial community); clarify the initial state of the degradation samples (e.g., size, weight), and supplement the number of parallel experiments to ensure the reliability of the data.

<u>A10:</u> Thank you for your insightful comments. PBS and PLA are well-recognized biodegradable polymers[1-2]. To evaluate the degradation behavior of TSFM fabricated from these bio-polymers under real natural conditions, the above fibrous samples were randomly buried in soil on the campus of Fuzhou University for natural degradation. As shown in Fig. 6g, the degradation of TSFM is a long process exceeding six months, during which the soil's physical and chemical properties are constantly undergoing dynamic changes due to seasonal and climatic variations. Consequently, the specific physicochemical properties of the soil cannot be provided. The detailed information regarding the degradation samples has been supplemented in the Methods section.

[1] Wang, CM, et al. Spider silk-inspired spinning of physically crosslinked ultrafine and electroactive poly(lactic acid) nanofibers for high-humidity PM filtration and intelligent respiratory diagnosis. *Chemical Engineering Journal* 530, 173106 (2026).

[2] David L. Biodegradable polyester-polybutylene succinate (PBS): a review. *Polymer Bulletin* 81, 5703-5752 (2024).
